# Supplementary material for: An umbrella review of the evidence linking oral health and systemic noncommunicable diseases
Source: Nat Commun. 2022 Dec 9;13:7614. doi: 10.1038/s41467-022-35337-8 (PMC9734115; doi:10.1038/s41467-022-35337-8)
Supplement: Supplementary file 2 — Description of Additional Supplementary Files [file 41467_2022_35337_MOESM2_ESM.pdf]

Description of Additional Supplementary Files

File Name: Supplementary Data 1
Description: List of excluded studies with justification for exclusion.

File Name: Supplementary Data 2
Description: Detailed information on the characteristics of the included systematic reviews.

File Name: Supplementary Data 3
Description: Summary of the country origin of the systematic review.

File Name: Supplementary Data 4
Description: AMSTAR 2 results.

File Name: Supplementary Data 5
Description: Detailed information on meta-analytical data and analysis of the included systematic reviews.

File Name: Supplementary Data 6
Description: PRISMA checklist.
