# Supplementary material for: An umbrella review of the evidence linking oral health and systemic noncommunicable diseases
Source: Nat Commun. 2022 Dec 9;13:7614. doi: 10.1038/s41467-022-35337-8 (PMC9734115; doi:10.1038/s41467-022-35337-8)
Supplement: Supplementary file 5 — Supplementary Data 3 [file 41467_2022_35337_MOESM5_ESM.docx]

| n | Country | References |  |
| --- | --- | --- | --- |
| 93 | China | 3, 7, 30, 31, 32, 34, 41, 44, 45, 48, 50, 53, 55, 59, 66, 85, 90, 108, 109, 113, 126, 131, 132, 145, 148, 149, 156, 162, 164, 165, 167, 168, 172, 175, 176, 177, 180, 194-196, 203, 204, 214, 215, 217, 220, 221, 224-228, 231, 233, 235, 236, 239, 244, 246-253, 255, 257, 258, 260-262, 265-268, 270-276, 278-285, 288, 291 | |
| 47 | Brazil | 5, 9, 16, 24, 27, 40, 43, 60, 62, 67, 68, 69, 71, 73, 79, 81, 84, 86, 91, 95, 96, 98, 99, 103, 112, 114, 124, 138, 139, 159, 161, 170, 179, 185, 187, 190-192, 197, 205, 208, 209, 213, 219, 256, 263, 286 |  |
| 22 | USA | 26, 35, 82, 115, 116, 134, 136, 144, 150-152, 158, 188, 189, 193, 200, 207, 230, 232, 234, 238, 292 | |
| 21 | United Kingdom | 21, 25, 36, 38, 39, 74-76, 78, 88, 102, 153, 155, 160, 166, 171, 182, 210, 289, 291, 293 |  |
| 15 | Spain | 20, 46, 65, 72, 87, 101, 117, 122, 123, 125, 135, 141, 143, 169, 268 |  |
| 13 | Italy | 1, 47, 49, 52, 58, 106, 127, 133, 163, 211, 223, 229, 243 |  |
| 10 | Australia | 6, 22, 23, 42, 111, 121, 142, 206, 237, 240 |  |
| 8 | The Netherlands | 13, 61, 63, 97, 118, 120, 146, 184 |  |
| 8 | Portugal | 4, 80, 89, 119, 130, 140, 178, 241 |  |
| 5 | France | 28, 56, 93, 129, 199 |  |
| 5 | Iran | 183, 186, 218, 242, 262 | |
| 5 | India | 12, 92, 110, 202, 285 |  |
| 4 | Germany | 15, 64, 94, 154 |  |
| 4 | Hong Kong | 10, 17, 19, 70 |  |
| 3 | Malaysia | 33, 51, 181 |  |
| 3 | Saudi Arabia | 147, 157, 201 |  |
| 3 | Switzerland | 14, 105, 173 |  |
| 3 | Turkey | 174, 216, 222 | |
| 2 | Canada | 77, 100 |  |
| 2 | Colombia | 54,198 |  |
| 2 | Norway | 2, 8 |  |
| 2 | Romania | 11, 29 |  |
| 2 | Greece | 137, 245 | |
| 2 | Taiwan | 254, 288 | |
| 1 | Belgium | 83 |  |
| 1 | Denmark | 18 |  |
| 1 | Ethiopia | 37 |  |
| 1 | Hungary | 212 |  |
| 1 | Lebanon | 104 |  |
| 1 | Morocco | 128 |  |
| 1 | New Zealand | 57 |  |
| 1 | Sweden | 107 |  |
| 1 | Indonesia | 276 | |

References

1. Bensi, C., Costacurta, M. & Docimo, R. Oral health in children with cerebral palsy: A systematic review and meta-analysis. *Spec. Care Dent. Off. Publ. Am. Assoc. Hosp. Dent. Acad. Dent. Handicap. Am. Soc. Geriatr. Dent.* **40**, 401–411 (2020).

2. Wagle, M. *et al.* Dental caries and preterm birth: a systematic review and meta-analysis. *BMJ Open* **8**, e018556 (2018).

3. Yang, M. *et al.* Poor oral health in patients with schizophrenia: A systematic review and meta-analysis. *Schizophr. Res.* **201**, 3–9 (2018).

4. Coelho, A. S. *et al.* Dental caries, diabetes mellitus, metabolic control and diabetes duration: A systematic review and meta-analysis. *J. Esthet. Restor. Dent. Off. Publ. Am. Acad. Esthet. Dent. Al* **32**, 291–309 (2020).

5. de Lima, A. K. A., Amorim Dos Santos, J., Stefani, C. M., Almeida de Lima, A. de & Damé-Teixeira, N. Diabetes mellitus and poor glycemic control increase the occurrence of coronal and root caries: a systematic review and meta-analysis. *Clin. Oral Investig.* **24**, 3801–3812 (2020).

6. Kisely, S., Baghaie, H., Lalloo, R., Siskind, D. & Johnson, N. W. A systematic review and meta-analysis of the association between poor oral health and severe mental illness. *Psychosom. Med.* **77**, 83–92 (2015).

7. Liu, T., Wei, Y., Zhu, Y. & Yang, W. Caries Status and Salivary Alterations of Type-1 Diabetes Mellitus in Children and Adolescents: A Systematic Review and Meta-analysis. *J. Evid. Based Dent. Pract.* **21**, 101496 (2021).

8. Skeie, M. S. *et al.* Oral health in children and adolescents with juvenile idiopathic arthritis – a systematic review and meta-analysis. *BMC Oral Health* **19**, 285 (2019).

9. Cademartori, M. G., Gastal, M. T., Nascimento, G. G., Demarco, F. F. & Corrêa, M. B. Is depression associated with oral health outcomes in adults and elders? A systematic review and meta-analysis. *Clin. Oral Investig.* **22**, 2685–2702 (2018).

10. Zhou, N., Wong, H. M., Wen, Y. F. & Mcgrath, C. Oral health status of children and adolescents with intellectual disabilities: a systematic review and meta‐analysis. *Dev. Med. Child Neurol.* **59**, 1019–1026 (2017).

11. Didilescu, A. C., Lazu, A., Pronk, C., Vacaru, R. P. & Brand, H. S. Clinical periodontal and dental findings in liver transplant patients: a systematic review and meta-analysis. *Br. Dent. J.* **228**, 108–116 (2020).

12. Mahajan, S., Bhaskar, N., Kaur, R. K. & Jain, A. A comparison of oral health status in diabetic and non-diabetic patients receiving hemodialysis – A systematic review and meta-analysis. *Diabetes Metab. Syndr. Clin. Res. Rev.* **15**, 102256 (2021).

13. Beukers, N. G. F. M., Su, N., Loos, B. G. & van der Heijden, G. J. M. G. Lower Number of Teeth Is Related to Higher Risks for ACVD and Death—Systematic Review and Meta-Analyses of Survival Data. *Front. Cardiovasc. Med.* **8**, 621626 (2021).

14. Papageorgiou, S. N. *et al.* Inflammatory bowel disease and oral health: systematic review and a meta-analysis. *J. Clin. Periodontol.* **44**, 382–393 (2017).

15. Akcalı, A., Yıldız, M. S., Akcalı, Z., Huck, O. & Friedmann, A. Periodontal condition of patients with Thalassemia Major: A systematic review and meta-analysis. *Arch. Oral Biol.* **102**, 113–121 (2019).

16. Cerutti-Kopplin, D. *et al.* Tooth Loss Increases the Risk of Diminished Cognitive Function: A Systematic Review and Meta-analysis. *JDR Clin. Transl. Res.* **1**, 10–19 (2016).

17. Dai, R. *et al.* A systematic review and meta-analysis of clinical, microbiological, and behavioural aspects of oral health among patients with stroke. *J. Dent.* **43**, 171–180 (2015).

18. Nascimento, G. G., Leite, F. R. M., Vestergaard, P., Scheutz, F. & López, R. Does diabetes increase the risk of periodontitis? A systematic review and meta-regression analysis of longitudinal prospective studies. *Acta Diabetol.* **55**, 653–667 (2018).

19. Li, L.-W., Wong, H. M., Sun, L., Wen, Y. F. & McGrath, C. P. Anthropometric Measurements and Periodontal Diseases in Children and Adolescents: A Systematic Review and Meta-Analysis. *Adv. Nutr.* **6**, 828–841 (2015).

20. Galletti, C., Camps-Font, O., Teixido-Tura, G., Llobet-Poal, I. & Gay-Escoda, C. Association between marfan syndrome and oral health status: A systematic review and meta-analysis. *Med. Oral Patol. Oral Cirugia Bucal* 0–0 (2019) doi:10.4317/medoral.23037.

21. Muñoz Aguilera, E. *et al.* Periodontitis is associated with hypertension: a systematic review and meta-analysis. *Cardiovasc. Res.* **116**, 28–39 (2020).

22. Kisely, S., Baghaie, H., Lalloo, R. & Johnson, N. W. Association between poor oral health and eating disorders: Systematic review and meta-analysis. *Br. J. Psychiatry* **207**, 299–305 (2015).

23. Kapellas, K. *et al.* Periodontal and chronic kidney disease association: A systematic review and meta-analysis: Periodontal and chronic kidney disease. *Nephrology* **24**, 202–212 (2019).

24. Nascimento, G. G. *et al.* Is weight gain associated with the incidence of periodontitis? A systematic review and meta-analysis. *J. Clin. Periodontol.* **42**, 495–505 (2015).

25. Nadim, R. *et al.* Influence of periodontal disease on risk of dementia: a systematic literature review and a meta-analysis. *Eur. J. Epidemiol.* **35**, 821–833 (2020).

26. Ioannidou, E., Malekzadeh, T. & Dongari-Bagtzoglou, A. Effect of Periodontal Treatment on Serum C-Reactive Protein Levels: A Systematic Review and Meta-Analysis. *J. Periodontol.* **77**, 1635–1642 (2006).

27. Jerônimo, L. S., Abreu, L. G., Cunha, F. A. & Esteves Lima, R. P. Association Between Periodontitis and Nosocomial Pneumonia: A Systematic Review and Meta-analysis of Observational Studies. *Oral Health Prev. Dent.* **18**, 11–17 (2020).

28. Darnaud, C. *et al.* Association between periodontitis and pulse wave velocity: a systematic review and meta-analysis. *Clin. Oral Investig.* **25**, 393–405 (2021).

29. Didilescu, A. C. *et al.* Oral diseases after liver transplantation: a systematic review and meta-analysis. *Br. Dent. J.* **231**, 117–124 (2021).

30. Gobin, R., Tian, D., Liu, Q. & Wang, J. Periodontal Diseases and the Risk of Metabolic Syndrome: An Updated Systematic Review and Meta-Analysis. *Front. Endocrinol.* **11**, 336 (2020).

31. Wei, Y., Zhong, Y., Wang, Y. & Huang, R. Association between periodontal disease and prostate cancer: a systematic review and meta-analysis. *Med. Oral Patol. Oral Cirugia Bucal* e459–e465 (2021) doi:10.4317/medoral.24308.

32. Guo, Z. *et al.* Periodontal disease and the risk of prostate cancer: a meta-analysis of cohort studies. *Int. Braz. J. Urol.* **47**, 1120–1130 (2021).

33. Akram, Z., Abduljabbar, T., Abu Hassan, M. I., Javed, F. & Vohra, F. Cytokine Profile in Chronic Periodontitis Patients with and without Obesity: A Systematic Review and Meta-Analysis. *Dis. Markers* **2016**, 4801418 (2016).

34. Zhu, J. *et al.* Association of circulating leptin and adiponectin with periodontitis: a systematic review and meta-analysis. *BMC Oral Health* **17**, 104 (2017).

35. Qi, X., Zhu, Z., Plassman, B. L. & Wu, B. Dose-Response Meta-Analysis on Tooth Loss With the Risk of Cognitive Impairment and Dementia. *J. Am. Med. Dir. Assoc.* **22**, 2039–2045 (2021).

36. Jordão, H. W. T., Coleman, H. G., Kunzmann, A. T. & McKenna, G. The association between erosive toothwear and gastro-oesophageal reflux-related symptoms and disease: A systematic review and meta-analysis. *J. Dent.* **95**, 103284 (2020).

37. Teshome, A. & Yitayeh, A. The effect of periodontal therapy on glycemic control and fasting plasma glucose level in type 2 diabetic patients: systematic review and meta-analysis. *BMC Oral Health* **17**, 31 (2017).

38. Simpson, T. C. *et al.* Treatment of periodontal disease for glycaemic control in people with diabetes mellitus. *Cochrane Database Syst. Rev.* (2015) doi:10.1002/14651858.CD004714.pub3.

39. Iheozor-Ejiofor, Z., Middleton, P., Esposito, M. & Glenny, A.-M. Treating periodontal disease for preventing adverse birth outcomes in pregnant women. *Cochrane Database Syst. Rev.* **2017**, (2017).

40. Galdino, T. M. *et al.* Periodontal Outcomes in Liver Transplantation Individuals: A Systematic Review and Meta-analysis. *J. Gastrointestin. Liver Dis.* **30**, 122–131 (2021).

41. Chen, H., Nie, S., Zhu, Y. & Lu, M. Teeth loss, teeth brushing and esophageal carcinoma: a systematic review and meta-analysis. *Sci. Rep.* **5**, 15203 (2015).

42. Ali, A., Lassi, Z. S., Kapellas, K., Jamieson, L. & Rumbold, A. R. A systematic review and meta-analysis of the association between periodontitis and oral high-risk human papillomavirus infection. *J. Public Health* **43**, e610–e619 (2021).

43. Nascimento, G. G. *et al.* Is there a relationship between obesity and tooth loss and edentulism? A systematic review and meta-analysis: Obesity and tooth loss. *Obes. Rev.* **17**, 587–598 (2016).

44. Yue, H. *et al.* Effects of non-surgical periodontal therapy on systemic inflammation and metabolic markers in patients undergoing haemodialysis and/or peritoneal dialysis: a systematic review and meta-analysis. *BMC Oral Health* **20**, 18 (2020).

45. Wang, J. *et al.* Relationship between periodontal disease and lung cancer: A systematic review and meta‐analysis. *J. Periodontal Res.* **55**, 581–593 (2020).

46. Otero Rey, E. M., Yáñez‐Busto, A., Rosa Henriques, I. F., López‐López, J. & Blanco‐Carrión, A. Lichen planus and diabetes mellitus: Systematic review and meta‐analysis. *Oral Dis.* **25**, 1253–1264 (2019).

47. Dioguardi, M. *et al.* The Association between Tooth Loss and Alzheimer’s Disease: a Systematic Review with Meta-Analysis of Case Control Studies. *Dent. J.* **7**, 49 (2019).

48. Cao, R. *et al.* Effect of non-surgical periodontal therapy on glycemic control of type 2 diabetes mellitus: a systematic review and Bayesian network meta-analysis. *BMC Oral Health* **19**, 176 (2019).

49. Corbella, S., Francetti, L., Taschieri, S., De Siena, F. & Fabbro, M. D. Effect of periodontal treatment on glycemic control of patients with diabetes: A systematic review and meta-analysis. *J. Diabetes Investig.* **4**, 502–509 (2013).

50. Wang, Q., Kang, J., Cai, X., Wu, Y. & Zhao, L. The association between chronic periodontitis and vasculogenic erectile dysfunction: a systematic review and meta-analysis. *J. Clin. Periodontol.* **43**, 206–215 (2016).

51. Gopinath, D., Kunnath Menon, R., K. Veettil, S., George Botelho, M. & Johnson, N. W. Periodontal Diseases as Putative Risk Factors for Head and Neck Cancer: Systematic Review and Meta-Analysis. *Cancers* **12**, 1893 (2020).

52. Rapone, B. *et al.* Does Periodontal Inflammation Affect Type 1 Diabetes in Childhood and Adolescence? A Meta-Analysis. *Front. Endocrinol.* **11**, 278 (2020).

53. Wu, C. *et al.* Epidemiologic relationship between periodontitis and type 2 diabetes mellitus. *BMC Oral Health* **20**, 204 (2020).

54. Botero, J. E., Rodríguez‐Medina, C., Jaramillo‐Echeverry, A. & Contreras, A. Association between human cytomegalovirus and periodontitis: A systematic review and meta‐analysis. *J. Periodontal Res.* **55**, 551–558 (2020).

55. Zhang, X., Gu, H., Xie, S. & Su, Y. Periodontitis in patients with psoriasis: A systematic review and meta‐analysis. *Oral Dis.* **28**, 33–43 (2022).

56. Schmitt, A., Carra, M. C., Boutouyrie, P. & Bouchard, P. Periodontitis and arterial stiffness: a systematic review and meta-analysis. *J. Clin. Periodontol.* **42**, 977–987 (2015).

57. Atieh, M. A., M. Faggion, C. & Seymour, G. J. Cytokines in patients with type 2 diabetes and chronic periodontitis: A systematic review and meta-analysis. *Diabetes Res. Clin. Pract.* **104**, e38–e45 (2014).

58. Adverse pregnancy outcomes and periodontitis: A systematic review and meta-analysis exploring potential association. *Quintessence Int.* **47**, 193–204 (2016).

59. Hua, F. *et al.* Oral hygiene care for critically ill patients to prevent ventilator-associated pneumonia. *Cochrane Database Syst. Rev.* (2016) doi:10.1002/14651858.CD008367.pub3.

60. Araújo, M. M. *et al.* Association between depression and periodontitis: a systematic review and meta-analysis. *J. Clin. Periodontol.* **43**, 216–228 (2016).

61. Ziukaite, L., Slot, D. E. & Van der Weijden, F. A. Prevalence of diabetes mellitus in people clinically diagnosed with periodontitis: A systematic review and meta-analysis of epidemiologic studies. *J. Clin. Periodontol.* **45**, 650–662 (2018).

62. Ferreira, M. K. M. *et al.* Is there an association between asthma and periodontal disease among adults? Systematic review and meta-analysis. *Life Sci.* **223**, 74–87 (2019).

63. Teeuw, W. J. *et al.* Treatment of periodontitis improves the atherosclerotic profile: a systematic review and meta-analysis. *J. Clin. Periodontol.* **41**, 70–79 (2014).

64. Papageorgiou, S. N., Reichert, C., Jäger, A. & Deschner, J. Effect of overweight/obesity on response to periodontal treatment: systematic review and a meta-analysis. *J. Clin. Periodontol.* **42**, 247–261 (2015).

65. Tomás, I., Diz, P., Tobías, A., Scully, C. & Donos, N. Periodontal health status and bacteraemia from daily oral activities: systematic review/meta-analysis. *J. Clin. Periodontol.* **39**, 213–228 (2012).

66. Peng, J. *et al.* The relationship between tooth loss and mortality from all causes, cardiovascular diseases, and coronary heart disease in the general population: systematic review and dose–response meta-analysis of prospective cohort studies. *Biosci. Rep.* **39**, BSR20181773 (2019).

67. Ferreira, R. de O. *et al.* Physical Activity Reduces the Prevalence of Periodontal Disease: Systematic Review and Meta-Analysis. *Front. Physiol.* **10**, 234 (2019).

68. da Silva, T. A., Abreu, L. G. & Esteves Lima, R. P. A meta‐analysis on the effect of periodontal treatment on the glomerular filtration rate of chronic kidney disease individuals: A systematic review and meta‐analysis was conducted to assess the impact of the periodontal treatment on the glomerular filtration rate of individuals with chronic kidney disease. *Spec. Care Dentist.* **41**, 670–678 (2021).

69. Daudt, L. D. *et al.* Association between metabolic syndrome and periodontitis: a systematic review and meta-analysis. *Braz. Oral Res.* **32**, (2018).

70. Zhao, D. *et al.* The directional and non-directional associations of periodontitis with chronic kidney disease: A systematic review and meta-analysis of observational studies. *J. Periodontal Res.* **53**, 682–704 (2018).

71. Moraschini, V., Barboza, E. S. P. & Peixoto, G. A. The impact of diabetes on dental implant failure: a systematic review and meta-analysis. *Int. J. Oral Maxillofac. Surg.* **45**, 1237–1245 (2016).

72. Leira, Y. *et al.* Is Periodontal Disease Associated with Alzheimer’s Disease? A Systematic Review with Meta-Analysis. *Neuroepidemiology* **48**, 21–31 (2017).

73. Moraschini, V., de Albuquerque Calasans-Maia, J. & Diuana Calasans-Maia, M. Association Between Asthma and Periodontal Disease: A Systematic Review and Meta-Analysis. *J. Periodontol.* 1–20 (2017) doi:10.1902/jop.2017.170363.

74. Joshi, C. *et al.* Detection of periodontal microorganisms in coronary atheromatous plaque specimens of myocardial infarction patients: A systematic review and meta-analysis. *Trends Cardiovasc. Med.* **31**, 69–82 (2021).

75. Conde-Agudelo, A., Villar, J. & Lindheimer, M. Maternal infection and risk of preeclampsia: Systematic review and metaanalysis. *Am. J. Obstet. Gynecol.* **198**, 7–22 (2008).

76. Simpson, T. C., Needleman, I., Wild, S. H., Moles, D. R. & Mills, E. J. Treatment of periodontal disease for glycaemic control in people with diabetes. in *Cochrane Database of Systematic Reviews* (ed. The Cochrane Collaboration) CD004714.pub2 (John Wiley & Sons, Ltd, 2010). doi:10.1002/14651858.CD004714.pub2.

77. Boutin, A. *et al.* Treatment of Periodontal Disease and Prevention of Preterm Birth: Systematic Review and Meta-analysis. *Am. J. Perinatol.* **30**, 537–544 (2012).

78. Suvan, J., D’Aiuto, F., Moles, D. R., Petrie, A. & Donos, N. Association between overweight/obesity and periodontitis in adults. A systematic review: Hypothalamic obesity. *Obes. Rev.* **12**, e381–e404 (2011).

79. Moura-Grec, P. G. de, Marsicano, J. A., Carvalho, C. A. P. de & Sales-Peres, S. H. de C. Obesity and periodontitis: systematic review and meta-analysis. *Ciênc. Saúde Coletiva* **19**, 1763–1772 (2014).

80. Machado, V. *et al.* Periodontitis Impact in Interleukin-6 Serum Levels in Solid Organ Transplanted Patients: A Systematic Review and Meta-Analysis. *Diagnostics* **10**, 184 (2020).

81. de Oliveira Ferreira, R. *et al.* Does periodontitis represent a risk factor for rheumatoid arthritis? A systematic review and meta-analysis. *Ther. Adv. Musculoskelet. Dis.* **11**, 1759720X1985851 (2019).

82. Monje, A., Catena, A. & Borgnakke, W. S. Association between diabetes mellitus/hyperglycaemia and peri-implant diseases: Systematic review and meta-analysis. *J. Clin. Periodontol.* **44**, 636–648 (2017).

83. Martens, L., De Smet, S., Yusof, M. Y. P. M. & Rajasekharan, S. Association between overweight/obesity and periodontal disease in children and adolescents: a systematic review and meta-analysis. *Eur. Arch. Paediatr. Dent.* **18**, 69–82 (2017).

84. Esteves Lima, R. P. *et al.* Association Between Periodontitis and Gestational Diabetes Mellitus: Systematic Review and Meta-Analysis. *J. Periodontol.* **87**, 48–57 (2016).

85. Li, W. *et al.* Is periodontal disease a risk indicator for colorectal cancer? A systematic review and meta‐analysis. *J. Clin. Periodontol.* **48**, 336–347 (2021).

86. Rosa, M. I. da, Pires, P. D. S., Medeiros, L. R., Edelweiss, M. I. & Martínez-Mesa, J. Periodontal disease treatment and risk of preterm birth: a systematic review and meta-analysis. *Cad. Saúde Pública* **28**, 1823–1833 (2012).

87. Leira, Y. *et al.* Association between periodontitis and ischemic stroke: a systematic review and meta-analysis. *Eur. J. Epidemiol.* **32**, 43–53 (2017).

88. Hussain, S. B. *et al.* Is there a bidirectional association between rheumatoid arthritis and periodontitis? A systematic review and meta-analysis. *Semin. Arthritis Rheum.* **50**, 414–422 (2020).

89. Botelho, J. *et al.* Stress, salivary cortisol and periodontitis: A systematic review and meta-analysis of observational studies. *Arch. Oral Biol.* **96**, 58–65 (2018).

90. Zheng, D. *et al.* Periodontal disease and emotional disorders: A meta‐analysis. *J. Clin. Periodontol.* **48**, 180–204 (2021).

91. Chambrone, L., Pannuti, C. M., Guglielmetti, M. R. & Chambrone, L. A. Evidence grade associating periodontitis with preterm birth and/or low birth weight: II. A systematic review of randomized trials evaluating the effects of periodontal treatment: Periodontitis and adverse pregnancy outcomes. *J. Clin. Periodontol.* **38**, 902–914 (2011).

92. Jain, A. *et al.* Effect of scaling and root planing as monotherapy on glycemic control in patients of Type 2 diabetes with chronic periodontitis: A systematic review and meta-analysis. *J. Indian Soc. Periodontol.* **23**, 303 (2019).

93. Martin-Cabezas, R. *et al.* Association between periodontitis and arterial hypertension: A systematic review and meta-analysis. *Am. Heart J.* **180**, 98–112 (2016).

94. Shang, R. & Gao, L. Impact of hyperglycemia on the rate of implant failure and peri-implant parameters in patients with type 2 diabetes mellitus. *J. Am. Dent. Assoc.* **152**, 189-201.e1 (2021).

95. Chambrone, L. *et al.* Periodontitis and chronic kidney disease: a systematic review of the association of diseases and the effect of periodontal treatment on estimated glomerular filtration rate. *J. Clin. Periodontol.* **40**, 443–456 (2013).

96. Chambrone, L., Guglielmetti, M. R., Pannuti, C. M. & Chambrone, L. A. Evidence grade associating periodontitis to preterm birth and/or low birth weight: I. A systematic review of prospective cohort studies: Periodontitis and adverse pregnancy outcomes. *J. Clin. Periodontol.* **38**, 795–808 (2011).

97. Paraskevas, S., Huizinga, J. D. & Loos, B. G. A systematic review and meta-analyses on C-reactive protein in relation to periodontitis. *J. Clin. Periodontol.* **35**, 277–290 (2008).

98. Fagundes, N. C. F. *et al.* Periodontitis As A Risk Factor For Stroke: A Systematic Review And Meta-Analysis. *Vasc. Health Risk Manag.* **Volume 15**, 519–532 (2019).

99. da Silva, H. E. C. *et al.* Effect of intra-pregnancy nonsurgical periodontal therapy on inflammatory biomarkers and adverse pregnancy outcomes: a systematic review with meta-analysis. *Syst. Rev.* **6**, 197 (2017).

100. Bi, W. G., Emami, E., Luo, Z.-C., Santamaria, C. & Wei, S. Q. Effect of periodontal treatment in pregnancy on perinatal outcomes: a systematic review and meta-analysis. *J. Matern.-Fetal Neonatal Med. Off. J. Eur. Assoc. Perinat. Med. Fed. Asia Ocean. Perinat. Soc. Int. Soc. Perinat. Obstet.* 1–10 (2019) doi:10.1080/14767058.2019.1678142.

101. Cabanillas‐Balsera, D. *et al.* Association between diabetes and nonretention of root filled teeth: a systematic review and meta‐analysis. *Int. Endod. J.* **52**, 297–306 (2019).

102. Nibali, L. *et al.* Association Between Metabolic Syndrome and Periodontitis: A Systematic Review and Meta-analysis. *J. Clin. Endocrinol. Metab.* **98**, 913–920 (2013).

103. Gomes‐Filho, I. S. *et al.* Periodontitis and respiratory diseases: A systematic review with meta‐analysis. *Oral Dis.* **26**, 439–446 (2020).

104. Kim, A. J., Lo, A. J., Pullin, D. A., Thornton-Johnson, D. S. & Karimbux, N. Y. Scaling and Root Planing Treatment for Periodontitis to Reduce Preterm Birth and Low Birth Weight: A Systematic Review and Meta-Analysis of Randomized Controlled Trials. *J. Periodontol.* **83**, 1508–1519 (2012).

105. Maldonado, A., Laugisch, O., Bürgin, W., Sculean, A. & Eick, S. Clinical periodontal variables in patients with and without dementia—a systematic review and meta-analysis. *Clin. Oral Investig.* **22**, 2463–2474 (2018).

106. Corbella, S. *et al.* Is periodontitis a risk indicator for cancer? A meta-analysis. *PLOS ONE* **13**, e0195683 (2018).

107. Chrcanovic, B. R., Albrektsson, T. & Wennerberg, A. Diabetes and Oral Implant Failure: A Systematic Review. *J. Dent. Res.* **93**, 859–867 (2014).

108. Tang, Q. *et al.* A Possible Link Between Rheumatoid Arthritis and Periodontitis: A Systematic Review and Meta-analysis. *Int. J. Periodontics Restorative Dent.* **37**, 79–86 (2017).

109. Deng, L., Li, C., Li, Q., Zhang, Y. & Zhao, H. [Periodontal treatment for cardiovascular risk factors: a systematic review]. *Hua Xi Kou Qiang Yi Xue Za Zhi Huaxi Kouqiang Yixue Zazhi West China J. Stomatol.* **31**, 463–467 (2013).

110. Gupta, A., Aggarwal, V., Mehta, N., Abraham, D. & Singh, A. Diabetes mellitus and the healing of periapical lesions in root filled teeth: a systematic review and meta‐analysis. *Int. Endod. J.* **53**, 1472–1484 (2020).

111. Kaur, S., Bright, R., Proudman, S. M. & Bartold, P. M. Does periodontal treatment influence clinical and biochemical measures for rheumatoid arthritis? A systematic review and meta-analysis. *Semin. Arthritis Rheum.* **44**, 113–122 (2014).

112. Gomes, M. S. *et al.* Can Apical Periodontitis Modify Systemic Levels of Inflammatory Markers? A Systematic Review and Meta-analysis. *J. Endod.* **39**, 1205–1217 (2013).

113. Wang, W. *et al.* Association between Periodontitis and Carotid Artery Calcification: A Systematic Review and Meta-Analysis. *BioMed Res. Int.* **2021**, 1–9 (2021).

114. Esteves Lima, R. P., Atanazio, A. R. S., Costa, F. O., Cunha, F. A. & Abreu, L. G. IMPACT OF NON-SURGICAL PERIODONTAL TREATMENT ON SERUM TNF-α LEVELS IN INDIVIDUALS WITH TYPE 2 DIABETES: A SYSTEMATIC REVIEW AND META-ANALYSIS. *J. Evid. Based Dent. Pract.* **21**, 101546 (2021).

115. Abariga, S. A. & Whitcomb, B. W. Periodontitis and gestational diabetes mellitus: a systematic review and meta-analysis of observational studies. *BMC Pregnancy Childbirth* **16**, 344 (2016).

116. Engebretson, S. & Kocher, T. Evidence that periodontal treatment improves diabetes outcomes: a systematic review and meta-analysis. *J. Periodontol.* **84**, S153–S163 (2013).

117. Figuero, E., Carrillo-de-Albornoz, A., Martín, C., Tobías, A. & Herrera, D. Effect of pregnancy on gingival inflammation in systemically healthy women: a systematic review. *J. Clin. Periodontol.* **40**, 457–473 (2013).

118. Kunnen, A. *et al.* Review Article: Periodontal disease and pre-eclampsia: a systematic review: Periodontal disease and pre-eclampsia. *J. Clin. Periodontol.* **37**, 1075–1087 (2010).

119. Machado, V., Escalda, C., Proença, L., Mendes, J. J. & Botelho, J. Is There a Bidirectional Association between Polycystic Ovarian Syndrome and Periodontitis? A Systematic Review and Meta-analysis. *J. Clin. Med.* **9**, E1961 (2020).

120. Georgiou, A. C., Crielaard, W., Armenis, I., de Vries, R. & van der Waal, S. V. Apical Periodontitis Is Associated with Elevated Concentrations of Inflammatory Mediators in Peripheral Blood: A Systematic Review and Meta-analysis. *J. Endod.* **45**, 1279-1295.e3 (2019).

121. Garde, S., Akhter, R., Nguyen, M. A., Chow, C. K. & Eberhard, J. Periodontal Therapy for Improving Lipid Profiles in Patients with Type 2 Diabetes Mellitus: A Systematic Review and Meta-Analysis. *Int. J. Mol. Sci.* **20**, 3826 (2019).

122. Lorenzo-Pouso, A. I., Castelo-Baz, P., Rodriguez-Zorrilla, S., Pérez-Sayáns, M. & Vega, P. Association between periodontal disease and inflammatory bowel disease: a systematic review and meta-analysis. *Acta Odontol. Scand.* **79**, 344–353 (2021).

123. Romandini, M. *et al.* Periodontitis, Edentulism, and Risk of Mortality: A Systematic Review with Meta-analyses. *J. Dent. Res.* **100**, 37–49 (2021).

124. Artese, H. P. C. *et al.* Periodontal Therapy and Systemic Inflammation in Type 2 Diabetes Mellitus: A Meta-Analysis. *PLOS ONE* **10**, e0128344 (2015).

125. Segura-Egea, J. J. *et al.* Association between diabetes and the prevalence of radiolucent periapical lesions in root-filled teeth: systematic review and meta-analysis. *Clin. Oral Investig.* **20**, 1133–1141 (2016).

126. Chen, Y. *et al.* Baseline HbA1c Level Influences the Effect of Periodontal Therapy on Glycemic Control in People with Type 2 Diabetes and Periodontitis: A Systematic Review on Randomized Controlled Trails. *Diabetes Ther.* **12**, 1249–1278 (2021).

127. Maisonneuve, P., Amar, S. & Lowenfels, A. B. Periodontal disease, edentulism, and pancreatic cancer: a meta-analysis. *Ann. Oncol. Off. J. Eur. Soc. Med. Oncol.* **28**, 985–995 (2017).

128. Bouziane, A., Ahid, S., Abouqal, R. & Ennibi, O. Effect of periodontal therapy on prevention of gastric *Helicobacter pylori* recurrence: a systematic review and meta-analysis. *J. Clin. Periodontol.* **39**, 1166–1173 (2012).

129. Darré, L., Vergnes, J.-N., Gourdy, P. & Sixou, M. Efficacy of periodontal treatment on glycaemic control in diabetic patients: A meta-analysis of interventional studies. *Diabetes Metab.* **34**, 497–506 (2008).

130. Machado, V. *et al.* Serum C-Reactive Protein and Periodontitis: A Systematic Review and Meta-Analysis. *Front. Immunol.* **12**, 706432 (2021).

131. Wu, Y. *et al.* Hematopoietic and lymphatic cancers in patients with periodontitis: a systematic review and meta-analysis. *Med. Oral Patol. Oral Cirugia Bucal* e21–e28 (2020) doi:10.4317/medoral.23166.

132. Lü, Z. *et al.* [Periodontal therapy for rheumatoid arthritis: a systematic review]. *Hua Xi Kou Qiang Yi Xue Za Zhi Huaxi Kouqiang Yixue Zazhi West China J. Stomatol.* **29**, 375–378 (2011).

133. Corbella, S., Taschieri, S., Francetti, L., De Siena, F. & Del Fabbro, M. Periodontal disease as a risk factor for adverse pregnancy outcomes: a systematic review and meta-analysis of case–control studies. *Odontology* **100**, 232–240 (2012).

134. Wijarnpreecha, K. *et al.* The Association between Periodontitis and Nonalcoholic Fatty Liver Disease: A Systematic Review and Meta-analysis. *J. Gastrointestin. Liver Dis.* **29**, 211–217 (2020).

135. Lorenzo‐Pouso, A. I. *et al.* Association between periodontitis and medication‐related osteonecrosis of the jaw: A systematic review and meta‐analysis. *J. Oral Pathol. Med.* **49**, 190–200 (2020).

136. Demmer, R. T. *et al.* The Influence of Anti-Infective Periodontal Treatment on C-Reactive Protein: A Systematic Review and Meta-Analysis of Randomized Controlled Trials. *PLoS ONE* **8**, e77441 (2013).

137. Polyzos, N. P. *et al.* Obstetric outcomes after treatment of periodontal disease during pregnancy: systematic review and meta-analysis. *BMJ* **341**, c7017–c7017 (2010).

138. Martorano-Fernandes, L. *et al.* Oral candidiasis and denture stomatitis in diabetic patients: Systematic review and meta-analysis. *Braz. Oral Res.* **34**, e113 (2020).

139. Peña, D. E. R., Innocentini, L. M. A. R., Saraiva, M. C. P., Lourenço, A. G. & Motta, A. C. F. Oral candidiasis prevalence in human immunodeficiency virus-1 and pulmonary tuberculosis coinfection: A systematic review and meta-analysis. *Microb. Pathog.* **150**, 104720 (2021).

140. Botelho, J. *et al.* Periodontitis and circulating blood cell profiles: a systematic review and meta-analysis. *Exp. Hematol.* **93**, 1–13 (2021).

141. Roca-Millan, E. *et al.* Periodontal treatment on patients with cardiovascular disease: Systematic review and meta-analysis. *Med. Oral Patol. Oral Cirugia Bucal* 0–0 (2018) doi:10.4317/medoral.22725.

142. Jensen, E., Allen, G., Bednarz, J., Couper, J. & Peña, A. Periodontal risk markers in children and adolescents with type 1 diabetes: A systematic review and meta‐analysis. *Diabetes Metab. Res. Rev.* **37**, (2021).

143. Manrique‐Corredor, E. J. *et al.* Maternal periodontitis and preterm birth: Systematic review and meta‐analysis. *Community Dent. Oral Epidemiol.* **47**, 243–251 (2019).

144. Ungprasert, P., Wijarnpreecha, K. & Wetter, D. A. Periodontitis and risk of psoriasis: a systematic review and meta-analysis. *J. Eur. Acad. Dermatol. Venereol.* **31**, 857–862 (2017).

145. Liu, L. H. *et al.* Chronic periodontitis and the risk of erectile dysfunction: a systematic review and meta-analysis. *Int. J. Impot. Res.* **29**, 43–48 (2017).

146. Teeuw, W. J., Gerdes, V. E. A. & Loos, B. G. Effect of Periodontal Treatment on Glycemic Control of Diabetic Patients. *Diabetes Care* **33**, 421–427 (2010).

147. Farook, F., Al Meshrafi, A., Mohamed Nizam, N. & Al Shammari, A. The Association Between Periodontitis and Erectile Dysfunction: A Systematic Review and Meta-Analysis. *Am. J. Mens Health* **15**, 155798832110072 (2021).

148. Wang, X., Han, X., Guo, X., Luo, X. & Wang, D. The Effect of Periodontal Treatment on Hemoglobin A1c Levels of Diabetic Patients: A Systematic Review and Meta-Analysis. *PLoS ONE* **9**, e108412 (2014).

149. Hu, X., Zhang, J., Qiu, Y. & Liu, Z. Periodontal disease and the risk of Alzheimer’s disease and mild cognitive impairment: a systematic review and meta‐analysis. *Psychogeriatrics* **21**, 813–825 (2021).

150. Schwartz, M., Acosta, L., Hung, Y.-L., Padilla, M. & Enciso, R. Effects of CPAP and mandibular advancement device treatment in obstructive sleep apnea patients: a systematic review and meta-analysis. *Sleep Breath.* **22**, 555–568 (2018).

151. Bahekar, A. A., Singh, S., Saha, S., Molnar, J. & Arora, R. The prevalence and incidence of coronary heart disease is significantly increased in periodontitis: A meta-analysis. *Am. Heart J.* **154**, 830–837 (2007).

152. Hsu, Y., Nair, M., Angelov, N., Lalla, E. & Lee, C. Impact of diabetes on clinical periodontal outcomes following non‐surgical periodontal therapy. *J. Clin. Periodontol.* **46**, 206–217 (2019).

153. Fuggle, N. R., Smith, T. O., Kaul, A. & Sofat, N. Hand to Mouth: A Systematic Review and Meta-Analysis of the Association between Rheumatoid Arthritis and Periodontitis. *Front. Immunol.* **7**, (2016).

154. Stöhr, J., Barbaresko, J., Neuenschwander, M. & Schlesinger, S. Bidirectional association between periodontal disease and diabetes mellitus: a systematic review and meta-analysis of cohort studies. *Sci. Rep.* **11**, 13686 (2021).

155. Ratz, T. *et al.* A possible link between ankylosing spondylitis and periodontitis: a systematic review and meta-analysis. *Rheumatology* **54**, 500–510 (2015).

156. Wu, D. *et al.* Decreased Hemoglobin Concentration and Iron Metabolism Disorder in Periodontitis: Systematic Review and Meta-Analysis. *Front. Physiol.* **10**, 1620 (2020).

157. Al-Jewair, T. S., Al-Jasser, R. & Almas, K. Periodontitis and obstructive sleep apnea’s bidirectional relationship: a systematic review and meta-analysis. *Sleep Breath.* **19**, 1111–1120 (2015).

158. Chaffee, B. W. & Weston, S. J. Association Between Chronic Periodontal Disease and Obesity: A Systematic Review and Meta-Analysis. *J. Periodontol.* **81**, 1708–1724 (2010).

159. da Silva, F. G., Pola, N. M., Casarin, M., Silva, C. F. e & Muniz, F. W. M. G. Association between clinical measures of gingival inflammation and obesity in adults: systematic review and meta-analyses. *Clin. Oral Investig.* **25**, 4281–4298 (2021).

160. Larvin, H., Kang, J., Aggarwal, V. R., Pavitt, S. & Wu, J. Risk of incident cardiovascular disease in people with periodontal disease: A systematic review and meta‐analysis. *Clin. Exp. Dent. Res.* **7**, 109–122 (2021).

161. Nepomuceno, R. *et al.* Serum lipid levels in patients with periodontal disease: A meta-analysis and meta-regression. *J. Clin. Periodontol.* **44**, 1192–1207 (2017).

162. Qiao, Y. *et al.* Rheumatoid arthritis risk in periodontitis patients: A systematic review and meta-analysis. *Joint Bone Spine* **87**, 556–564 (2020).

163. Dicembrini, I. *et al.* Type 1 diabetes and periodontitis: prevalence and periodontal destruction—a systematic review. *Acta Diabetol.* **57**, 1405–1412 (2020).

164. Xiao, L., Zhang, Q., Peng, Y., Wang, D. & Liu, Y. The effect of periodontal bacteria infection on incidence and prognosis of cancer: A systematic review and meta-analysis. *Medicine (Baltimore)* **99**, e19698 (2020).

165. Yang, S. *et al.* Association between periodontitis and peripheral artery disease: a systematic review and meta-analysis. *BMC Cardiovasc. Disord.* **18**, 141 (2018).

166. Orlandi, M. *et al.* Association between periodontal disease and its treatment, flow-mediated dilatation and carotid intima-media thickness: A systematic review and meta-analysis. *Atherosclerosis* **236**, 39–46 (2014).

167. Zhang, Y. *et al.* The Association between Periodontitis and Inflammatory Bowel Disease: A Systematic Review and Meta-analysis. *BioMed Res. Int.* **2021**, 1–8 (2021).

168. Zhang, J. *et al.* Influence of anti‐rheumatic agents on the periodontal condition of patients with rheumatoid arthritis and periodontitis: A systematic review and meta‐analysis. *J. Periodontal Res.* **56**, 1099–1115 (2021).

169. Moliner-Sánchez, C. A. *et al.* Effect of per Capita Income on the Relationship between Periodontal Disease during Pregnancy and the Risk of Preterm Birth and Low Birth Weight Newborn. Systematic Review and Meta-Analysis. *Int. J. Environ. Res. Public. Health* **17**, 8015 (2020).

170. Baeza, M. *et al.* Effect of periodontal treatment in patients with periodontitis and diabetes: systematic review and meta-analysis. *J. Appl. Oral Sci.* **28**, e20190248 (2020).

171. Rutter-Locher, Z., Smith, T. O., Giles, I. & Sofat, N. Association between Systemic Lupus Erythematosus and Periodontitis: A Systematic Review and Meta-analysis. *Front. Immunol.* **8**, 1295 (2017).

172. Chen, Y., Zhu, B., Wu, C., Lin, R. & Zhang, X. Periodontal Disease and Tooth Loss Are Associated with Lung Cancer Risk. *BioMed Res. Int.* **2020**, 1–12 (2020).

173. Koletsi, D., Iliadi, A., Tzanetakis, G. N., Vavuranakis, M. & Eliades, T. Cardiovascular Disease and Chronic Endodontic Infection. Is There an Association? A Systematic Review and Meta-Analysis. *Int. J. Environ. Res. Public. Health* **18**, 9111 (2021).

174. Ozturk, A. Periodontal Treatment Is Associated With Improvement in Gastric Helicobacter pylori Eradication: An Updated Meta-analysis of Clinical Trials. *Int. Dent. J.* **71**, 188–196 (2021).

175. Zhou, X., Cao, F., Lin, Z. & Wu, D. Updated Evidence of Association Between Periodontal Disease and Incident Erectile Dysfunction. *J. Sex. Med.* **16**, 61–69 (2019).

176. Lv, X. *et al.* Periodontal Disease and Age-Related Macular Degeneration: A Meta-Analysis of 112,240 Participants. *BioMed Res. Int.* **2020**, 1–11 (2020).

177. Xu, J. & Duan, X. Association between periodontitis and hyperlipidaemia: A systematic review and meta‐analysis. *Clin. Exp. Pharmacol. Physiol.* **47**, 1861–1873 (2020).

178. Silva, D. S. *et al.* Evidence‐Based Research on Effectiveness of Periodontal Treatment in Rheumatoid Arthritis Patients: A Systematic Review and Meta‐Analysis. *Arthritis Care Res.* **74**, 1723–1735 (2022).

179. Calderaro, D. C. *et al.* Influência do tratamento periodontal na artrite reumatoide: revisão sistemática e metanálise. *Rev. Bras. Reumatol.* **57**, 238–244 (2017).

180. Ren, Q., Yan, X., Zhou, Y. & Li, W. X. Periodontal therapy as adjunctive treatment for gastric *Helicobacter pylori* infection. *Cochrane Database Syst. Rev.* **2016**, (2016).

181. Zainal Abidin, Z. *et al.* Periodontal health status of children and adolescents with diabetes mellitus: a systematic review and meta‐analysis. *Aust. Dent. J.* **66**, (2021).

182. Hussain, S. B. *et al.* Periodontitis and Systemic Lupus Erythematosus: A systematic review and meta‐analysis. *J. Periodontal Res.* **57**, 1–10 (2022).

183. Mirzaei, A. *et al.* Association of hyperglycaemia and periodontitis: an updated systematic review and meta-analysis. *J. Diabetes Metab. Disord.* **20**, 1327–1336 (2021).

184. Maarse, F. *et al.* Sjögren’s syndrome is not a risk factor for periodontal disease: a systematic review. *Clin. Exp. Rheumatol.* **37 Suppl 118**, 225–233 (2019).

185. Souza, M. L., Massignan, C., Glazer Peres, K. & Aurélio Peres, M. Association between metabolic syndrome and tooth loss. *J. Am. Dent. Assoc.* **150**, 1027-1039.e7 (2019).

186. Jalili, M., Mahmoodabadi, K. A. & Sayehmiri, K. Relationship between Helicobacter pylori and Periodontal Diseases: A Meta-Analysis Study and Systematic Review. *Open Dent. J.* **14**, 362–368 (2020).

187. Silveira, A. L. N. de M. e S., Magno, M. B. & Soares, T. R. C. The relationship between special needs and dental trauma. A systematic review and meta‐analysis. *Dent. Traumatol.* **36**, 218–236 (2020).

188. Aminoshariae, A., Kulild, J. & Fouad, A. F. The impact of cardiovascular disease and endodontic outcome: a systematic review of longitudinal studies. *Clin. Oral Investig.* **24**, 3813–3819 (2020).

189. Aminoshariae, A., Kulild, J. C. & Fouad, A. F. The Impact of Endodontic Infections on the Pathogenesis of Cardiovascular Disease(s): A Systematic Review with Meta-analysis Using GRADE. *J. Endod.* **44**, 1361-1366.e3 (2018).

190. Alvarenga, M. O. P. *et al.* Masticatory Dysfunction by Extensive Tooth Loss as a Risk Factor for Cognitive Deficit: A Systematic Review and Meta-Analysis. *Front. Physiol.* **10**, 832 (2019).

191. Araújo, B. C. L., de Magalhães Simões, S., de Gois-Santos, V. T. & Martins-Filho, P. R. S. Association Between Mouth Breathing and Asthma: a Systematic Review and Meta-analysis. *Curr. Allergy Asthma Rep.* **20**, 24 (2020).

192. Hermont, A. P. *et al.* Tooth Erosion and Eating Disorders: A Systematic Review and Meta-Analysis. *PLoS ONE* **9**, e111123 (2014).

193. Lockhart, P. B. *et al.* Effect of dental treatment before cardiac valve surgery. *J. Am. Dent. Assoc.* **150**, 739-747.e9 (2019).

194. Wang, T. *et al.* Periodontal disease and cognitive deficits: A systematic review and meta-analysis. *Neurol. Asia* 12 (2020).

195. Jiang, X., Zhu, Y., Liu, Z., Tian, Z. & Zhu, S. Association between diabetes and dental implant complications: a systematic review and meta-analysis. *Acta Odontol. Scand.* **79**, 9–18 (2021).

196. Fang, W. *et al.* Tooth loss as a risk factor for dementia: systematic review and meta-analysis of 21 observational studies. *BMC Psychiatry* **18**, 345 (2018).

197. Souto-Souza, D. *et al.* Is there an association between attention deficit hyperactivity disorder in children and adolescents and the occurrence of bruxism? A systematic review and meta-analysis. *Sleep Med. Rev.* **53**, 101330 (2020).

198. Ríos-Osorio, N. *et al.* Association between type 2 diabetes mellitus and the evolution of endodontic pathology. *Quintessence Int. Berl. Ger. 1985* **51**, 100–107 (2020).

199. Blaizot, A., Vergnes, J.-N., Nuwwareh, S., Amar, J. & Sixou, M. Periodontal diseases and cardiovascular events: meta-analysis of observational studies. *Int. Dent. J.* **59**, 197–209 (2009).

200. Oh, B. *et al.* Association between residual teeth number in later life and incidence of dementia: A systematic review and meta-analysis. *BMC Geriatr.* **18**, 48 (2018).

201. AlOtaibi, A., Ben Shaber, S., AlBatli, A., AlGhamdi, T. & Murshid, E. A systematic review of population-based gingival health studies among children and adolescents with autism spectrum disorder. *Saudi Dent. J.* **33**, 370–374 (2021).

202. Easwaran, H. N. *et al.* Early Childhood Caries and Iron Deficiency Anaemia: A Systematic Review and Meta-Analysis. *Caries Res.* **56**, 36–46 (2022).

203. Ji, S.-Q. *et al.* Iron deficiency and early childhood caries: a systematic review and meta-analysis. *Chin. Med. J. (Engl.)* **134**, 2832–2837 (2021).

204. Sun, X.-N., Zhou, J.-B. & Li, N. Poor Oral Health in Patients with Schizophrenia: a Meta-Analysis of Case-Control Studies. *Psychiatr. Q.* **92**, 135–145 (2021).

205. Drumond, V. Z. *et al.* Dental Caries in Children with Attention Deficit/Hyperactivity Disorder: A Meta-Analysis. *Caries Res.* **56**, 3–14 (2022).

206. Le, Q.-A. *et al.* Does Treatment of Gingivitis During Pregnancy Improve Pregnancy Outcomes? A Systematic Review and Meta-Analysis. *Oral Health Prev. Dent.* **19**, 565–572 (2021).

207. Marzouk, T. *et al.* Association between oral clefts and periodontal clinical measures: A meta‐analysis. *Int. J. Paediatr. Dent.* **32**, 558–575 (2022).

208. Del Rei Daltro Rosa, C. D. *et al.* Does non-surgical periodontal treatment influence on rheumatoid arthritis? A systematic review and meta-analysis. *Saudi Dent. J.* **33**, 795–804 (2021).

209. Porto, E. C. L. *et al.* Periodontite materna e baixo peso ao nascer: revisão sistemática e metanálise. *Ciênc. Saúde Coletiva* **26**, 5383–5392 (2021).

210. Orlandi, M. *et al.* Impact of the treatment of periodontitis on systemic health and quality of life: A systematic review. *J. Clin. Periodontol.* jcpe.13554 (2021) doi:10.1111/jcpe.13554.

211. Serni, L. *et al.* Association between chronic kidney disease and periodontitis. A systematic review and metanalysis. *Oral Dis.* odi.14062 (2021) doi:10.1111/odi.14062.

212. Andrade, C. A. S. *et al.* Survival rate and peri-implant evaluation of immediately loaded dental implants in individuals with type 2 diabetes mellitus: a systematic review and meta-analysis. *Clin. Oral Investig.* **26**, 1797–1810 (2022).

213. Gusman, D. J. R. *et al.* Periodontal disease severity in subjects with dementia: A systematic review and meta-analysis. *Arch. Gerontol. Geriatr.* **76**, 147–159 (2018).

214. Pi, X. *et al.* A Meta-Analysis of Oral Health Status of Children with Autism. *J. Clin. Pediatr. Dent.* **44**, 1–7 (2020).

215. Zhang, Y., Lin, L., Liu, J., Shi, L. & Lu, J. Dental Caries Status in Autistic Children: A Meta-analysis. *J. Autism Dev. Disord.* **50**, 1249–1257 (2020).

216. Hatipoğlu, Ö., Önsüren, A. S., Hatipoğlu, F. P. & Kurt, A. Caries‐related salivary parameters and oral microbial flora in patients with type 1 diabetes: A meta‐analysis. *Diabetes Metab. Res. Rev.* **38**, (2022).

217. Xi, W. *et al.* [Oral health status of patients undergoing hemodialysis: a Meta-analysis]. *Hua Xi Kou Qiang Yi Xue Za Zhi Huaxi Kouqiang Yixue Zazhi West China J. Stomatol.* **35**, 155–161 (2017).

218. Sharifi, R. *et al.* Evaluation of Serum and Salivary Iron and Ferritin Levels in Children with Dental Caries: A Meta-Analysis and Trial Sequential Analysis. *Children* **8**, 1034 (2021).

219. Granja, G. L. *et al.* Occurrence of bruxism in individuals with autism spectrum disorder: A systematic review and meta‐analysis. *Spec. Care Dentist.* **42**, 476–485 (2022).

220. Xu, S. *et al.* The association between periodontal disease and the risk of myocardial infarction: a pooled analysis of observational studies. *BMC Cardiovasc. Disord.* **17**, 50 (2017).

221. Zeng, L.-N. *et al.* Oral health in patients with stroke: a meta-analysis of comparative studies. *Top. Stroke Rehabil.* **27**, 75–80 (2020).

222. Hatipoğlu, Ö. & Pertek Hatipoğlu, F. Association between asthma and caries-related salivary factors: a meta-analysis. *J. Asthma Off. J. Assoc. Care Asthma* **59**, 38–53 (2022).

223. Arduino, P. G., Cabras, M., Lodi, G. & Petti, S. Herpes simplex virus type 1 in subgingival plaque and periodontal diseases. Meta‐analysis of observational studies. *J. Periodontal Res.* **57**, 256–268 (2022).

224. Lianhui, Y., Meifei, L., Zhongyue, H. & Yunzhi, F. [Association between chronic periodontitis and hyperlipidemia: a Meta-analysis based on observational studies]. *Hua Xi Kou Qiang Yi Xue Za Zhi Huaxi Kouqiang Yixue Zazhi West China J. Stomatol.* **35**, 419–426 (2017).

225. Shi, T. *et al.* Periodontal disease and susceptibility to breast cancer: A meta-analysis of observational studies. *J. Clin. Periodontol.* **45**, 1025–1033 (2018).

226. Zeng, X.-T. *et al.* Periodontal Disease and Risk of Head and Neck Cancer: A Meta-Analysis of Observational Studies. *PLoS ONE* **8**, e79017 (2013).

227. Zeng, L. *et al.* Oral health in patients with dementia: A meta‐analysis of comparative and observational studies. *Int. J. Geriatr. Psychiatry* **36**, 467–478 (2021).

228. Zeng, X.-T. *et al.* Periodontal Disease and Incident Lung Cancer Risk: A Meta-Analysis of Cohort Studies. *J. Periodontol.* **87**, 1158–1164 (2016).

229. Sgolastra, F., Petrucci, A., Severino, M., Gatto, R. & Monaco, A. Relationship between Periodontitis and Pre-Eclampsia: A Meta-Analysis. *PLoS ONE* **8**, e71387 (2013).

230. Uppal, A. *et al.* The Effectiveness of Periodontal Disease Treatment During Pregnancy in Reducing the Risk of Experiencing Preterm Birth and Low Birth Weight. *J. Am. Dent. Assoc.* **141**, 1423–1434 (2010).

231. Huang, X. *et al.* Maternal periodontal disease and risk of preeclampsia: A meta-analysis. *J. Huazhong Univ. Sci. Technolog. Med. Sci.* **34**, 729–735 (2014).

232. Liew, A., Punnanithinont, N., Lee, Y.-C. & Yang, J. Effect of non-surgical periodontal treatment on HbA1c: a meta-analysis of randomized controlled trials. *Aust. Dent. J.* **58**, 350–357 (2013).

233. Xuan, K., Jha, A. R., Zhao, T., Uy, J. P. & Sun, C. Is periodontal disease associated with increased risk of colorectal cancer? A meta‐analysis. *Int. J. Dent. Hyg.* **19**, 50–61 (2021).

234. Ren, H. G. *et al.* Oral health and risk of colorectal cancer: results from three cohort studies and a meta-analysis. *Ann. Oncol.* **27**, 1329–1336 (2016).

235. Xu, S., Zhang, G., Xia, C. & Tan, Y. Associations Between Poor Oral Health and Risk of Squamous Cell Carcinoma of the Head and Neck: A Meta-Analysis of Observational Studies. *J. Oral Maxillofac. Surg.* **77**, 2128–2142 (2019).

236. Qiu-Ying Sun *et al.* Effects of Periodontal Treatment on Glycemic Control in Type 2 Diabetic Patients: A Meta-Analysis of Randomized Controlled Trials. *Chin. J. Physiol.* **57**, (2014).

237. George, A. *et al.* Periodontal treatment during pregnancy and birth outcomes: a meta-analysis of randomised trials. *Int. J. Evid. Based Healthc.* **9**, 122–147 (2011).

238. Michaud, D. S., Fu, Z., Shi, J. & Chung, M. Periodontal Disease, Tooth Loss, and Cancer Risk. *Epidemiol. Rev.* **39**, 49–58 (2017).

239. Zhong, H.-J., Xie, H.-X., Luo, X.-M. & Zhang, E.-H. Association between periodontitis and systemic lupus erythematosus: a meta-analysis. *Lupus* **29**, 1189–1197 (2020).

240. Le, Q.-A. *et al.* DIFFERENTIAL IMPACT OF PERIODONTAL TREATMENT STRATEGIES DURING PREGNANCY ON PERINATAL OUTCOMES: A SYSTEMATIC REVIEW AND META-ANALYSIS. *J. Evid.-Based Dent. Pract.* **22**, 101666 (2022).

241. Noites, R., Teixeira, M., Cavero-Redondo, I., Alvarez-Bueno, C. & Ribeiro, F. Apical Periodontitis and Cardiovascular Disease in Adults: A Systematic Review with Meta-Analysis. *Rev. Cardiovasc. Med.* **23**, 0100 (2022).

242. Ahmadinia, A. R. *et al.* Association between type 2 diabetes (T2D) and tooth loss: a systematic review and meta-analysis. *BMC Endocr. Disord.* **22**, 100 (2022).

243. Sgolastra, F., Severino, M., Pietropaoli, D., Gatto, R. & Monaco, A. Effectiveness of Periodontal Treatment to Improve Metabolic Control in Patients With Chronic Periodontitis and Type 2 Diabetes: A Meta-Analysis of Randomized Clinical Trials. *J. Periodontol.* **84**, 958–973 (2013).

244. Ye, L., Jiang, Y., Liu, W. & Tao, H. Correlation between periodontal disease and oral cancer risk: A meta-analysis. *J. Cancer Res. Ther.* **12**, 237 (2016).

245. Polyzos, N. P. *et al.* Effect of periodontal disease treatment during pregnancy on preterm birth incidence: a metaanalysis of randomized trials. *Am. J. Obstet. Gynecol.* **200**, 225–232 (2009).

246. Wei, B.-J., Chen, Y.-J., Yu, L. & Wu, B. Periodontal Disease and Risk of Preeclampsia: A Meta-Analysis of Observational Studies. *PLoS ONE* **8**, e70901 (2013).

247. Yao, Q.-W., Zhou, D.-S., Peng, H.-J., Ji, P. & Liu, D.-S. Association of periodontal disease with oral cancer: a meta-analysis. *Tumor Biol.* **35**, 7073–7077 (2014).

248. Zhu, C. *et al.* Association between Herpesviruses and Chronic Periodontitis: A Meta-Analysis Based on Case-Control Studies. *PLOS ONE* **10**, e0144319 (2015).

249. Ma, P., Dai, S., Jin, C., Yao, Y. & Zou, C. Tooth loss and risk of colorectal cancer: a dose&ndash;response meta-analysis of prospective cohort studies. *OncoTargets Ther.* **Volume 11**, 1617–1623 (2018).

250. Li, F. *et al.* Herpesviruses in etiopathogenesis of aggressive periodontitis: A meta-analysis based on case-control studies. *PLOS ONE* **12**, e0186373 (2017).

251. Qin, X., Zhao, Y. & Guo, Y. Periodontal disease and myocardial infarction risk: A meta-analysis of cohort studies. *Am. J. Emerg. Med.* **48**, 103–109 (2021).

252. Zeng, X.-T. *et al.* Periodontal Disease and Risk of Chronic Obstructive Pulmonary Disease: A Meta-Analysis of Observational Studies. *PLoS ONE* **7**, e46508 (2012).

253. Zhang, Y. *et al.* Is periodontitis a risk indicator for gastrointestinal cancers? A meta‐analysis of cohort studies. *J. Clin. Periodontol.* **47**, 134–147 (2020).

254. Wang, T.-F., Jen, I.-A., Chou, C. & Lei, Y.-P. Effects of Periodontal Therapy on Metabolic Control in Patients With Type 2 Diabetes Mellitus and Periodontal Disease: A Meta-Analysis. *Medicine (Baltimore)* **93**, e292 (2014).

255. Liu, F. *et al.* A meta-analysis of emotional disorders as possible risk factors for chronic periodontitis. *Medicine (Baltimore)* **97**, e11434 (2018).

256. Rodrigues, R. P. C. B. *et al.* Salivary changes in chronic kidney disease and in patients undergoing hemodialysis: a systematic review and meta-analysis. *J. Nephrol.* **35**, 1339–1367 (2022).

257. Chen, Z. *et al.* A meta-analysis of the association between the presence of Helicobacter pylori and periodontal diseases. *Medicine (Baltimore)* **98**, e15922 (2019).

258. Liu, Z. *et al.* Systemic Oxidative Stress Biomarkers in Chronic Periodontitis: A Meta-Analysis. *Dis. Markers* **2014**, 1–10 (2014).

259. She, Y. *et al.* Periodontitis and inflammatory bowel disease: a meta-analysis. *BMC Oral Health* **20**, 67 (2020).

260. Zeng, X.-T. *et al.* Periodontal disease and carotid atherosclerosis: A meta-analysis of 17,330 participants. *Int. J. Cardiol.* **203**, 1044–1051 (2016).

261. Gao, Z., Lv, J. & Wang, M. Epstein–Barr virus is associated with periodontal diseases: A meta-analysis based on 21 case–control studies. *Medicine (Baltimore)* **96**, e5980 (2017).

262. Khodadadi, N., Khodadadi, M. & Zamani, M. Is periodontitis associated with obstructive sleep apnea? A systematic review and meta-analysis. *J. Clin. Exp. Dent.* e359–e365 (2022) doi:10.4317/jced.59478.

263. Foratori-Junior, G. A. *et al.* Is overweight associated with periodontitis in pregnant women? Systematic review and meta-analysis. *Jpn. Dent. Sci. Rev.* **58**, 41–51 (2022).

264. Zhang, S. *et al.* Oral manifestations of patients with systemic sclerosis: a meta-analysis for case-controlled studies. *BMC Oral Health* **21**, 250 (2021).

265. Ma, H., Zheng, J. & Li, X. Potential risk of certain cancers among patients with Periodontitis: a supplementary meta-analysis of a large-scale population. *Int. J. Med. Sci.* **17**, 2531–2543 (2020).

266. Sun, J. *et al.* Non-surgical periodontal treatment improves rheumatoid arthritis disease activity: a meta-analysis. *Clin. Oral Investig.* **25**, 4975–4985 (2021).

267. Li, Q. *et al.* Effect of non-surgical periodontal treatment on glycemic control of patients with diabetes: a meta-analysis of randomized controlled trials. *Trials* **16**, 291 (2015).

268. López-Valverde, N. *et al.* Possible Association of Periodontal Diseases With Helicobacter pylori Gastric Infection: A Systematic Review and Meta-Analysis. *Front. Med.* **9**, 822194 (2022).

269. Wei, X. *et al.* The association between chronic periodontitis and oral Helicobacter pylori: A meta-analysis. *PLOS ONE* **14**, e0225247 (2019).

270. Gao, S. *et al.* Periodontitis and Number of Teeth in the Risk of Coronary Heart Disease: An Updated Meta-Analysis. *Med. Sci. Monit.* **27**, (2021).

271. Zheng, M. *et al.* Prevalence of periodontitis in people clinically diagnosed with diabetes mellitus: a meta-analysis of epidemiologic studies. *Acta Diabetol.* **58**, 1307–1327 (2021).

272. Guo, H. *et al.* The Effect of Periodontitis on Dementia and Cognitive Impairment: A Meta-Analysis. *Int. J. Environ. Res. Public. Health* **18**, 6823 (2021).

273. Qiao, P. *et al.* Psoriasis Patients Suffer From Worse Periodontal Status—A Meta-Analysis. *Front. Med.* **6**, 212 (2019).

274. Huang, Y. *et al.* Effects of non-surgical periodontal therapy on periodontal clinical data in periodontitis patients with rheumatoid arthritis: a meta-analysis. *BMC Oral Health* **21**, 340 (2021).

275. Xu, S., Zhang, G., Guo, J. & Tan, Y. Associations between osteoporosis and risk of periodontitis: A pooled analysis of observational studies. *Oral Dis.* **27**, 357–369 (2021).

276. Maulani, C. *et al.* Association between Epstein-Barr virus and periodontitis: A meta-analysis. *PLOS ONE* **16**, e0258109 (2021).

277. Leng, W.-D., Zeng, X.-T., Kwong, J. S. W. & Hua, X.-P. Periodontal disease and risk of coronary heart disease: An updated meta-analysis of prospective cohort studies. *Int. J. Cardiol.* **201**, 469–472 (2015).

278. Zhang, J., Jiang, H., Sun, M. & Chen, J. Association between periodontal disease and mortality in people with CKD: a meta-analysis of cohort studies. *BMC Nephrol.* **18**, 269 (2017).

279. Shao, J. *et al.* Periodontal Disease and Breast Cancer: A Meta-Analysis of 1,73,162 Participants. *Front. Oncol.* **8**, 601 (2018).

280. Shi, Q. *et al.* Patients with Chronic Obstructive Pulmonary Disease Suffer from Worse Periodontal Health—Evidence from a Meta-Analysis. *Front. Physiol.* **9**, 33 (2018).

281. Qiu, C., Zhou, W., Shi, W. & Song, Z. Association between periodontitis and Alzheimer disease: a meta analysis. *Shanghai J. Stomatol.* **29**, 661–668 (2020).

282. Xie, W.-Z. *et al.* Periodontal Disease and Risk of Bladder Cancer: A Meta-Analysis of 298476 Participants. *Front. Physiol.* **9**, 979 (2018).

283. Wu, Z., Xiao, C., Chen, F., Wang, Y. & Guo, Z. Pulmonary disease and periodontal health: a meta-analysis. *Sleep Breath.* (2022) doi:10.1007/s11325-022-02577-3.

284. Chen, J. *et al.* Tooth Loss Is Associated With Increased Risk of Dementia and With a Dose-Response Relationship. *Front. Aging Neurosci.* **10**, 415 (2018).

285. Sayeed, G. & Varghese, S. Association between periodontitis and metabolic syndrome in females: A systematic review and meta-analysis. *J. Int. Soc. Prev. Community Dent.* **0**, 0 (2021).

286. França, L. F. de C. *et al.* Comparative analysis of blood parameters of the erythrocyte lineage between patients with chronic periodontitis and healthy patients: Results obtained from a meta-analysis. *Arch. Oral Biol.* **97**, 144–149 (2019).

287. Shi, Q. *et al.* Association between Myocardial Infarction and Periodontitis: A Meta-Analysis of Case-Control Studies. *Front. Physiol.* **7**, (2016).

288. Wu, S.-Y. *et al.* Periodontal conditions in patients with Sjögren’s syndrome: A meta-analysis. *J. Dent. Sci.* **16**, 1222–1232 (2021).

289. Simpson, T. C. *et al.* Treatment of periodontitis for glycaemic control in people with diabetes mellitus. *Cochrane Database Syst. Rev.* **2022**, (2022).

290. Luo, Y. *et al.* Effect of periodontal treatments on blood pressure. *Cochrane Database Syst. Rev.* **2021**, (2021).

291. Irwandi, R. A., Kuswandani, S. O., Harden, S., Marletta, D. & D’Aiuto, F. Circulating inflammatory cell profiling and periodontitis: A systematic review and meta‐analysis. *J. Leukoc. Biol.* **111**, 1069–1096 (2022).

292. Antonarakis, G. S., Palaska, P.-K. & Herzog, G. Caries prevalence in non-syndromic patients with cleft lip and/or palate: a meta-analysis. *Caries Res.* **47**, 406–413 (2013).

293. Grewcock, R. E., Innes, N. P. T., Mossey, P. A. & Robertson, M. D. Caries in children with and without orofacial clefting: A systematic review and meta-analysis. *Oral Dis.* **28**, 1400–1411 (2022).
