# Supplementary material for: An umbrella review of the evidence linking oral health and systemic noncommunicable diseases
Source: Nat Commun. 2022 Dec 9;13:7614. doi: 10.1038/s41467-022-35337-8 (PMC9734115; doi:10.1038/s41467-022-35337-8)
Supplement: Supplementary file 6 — Supplementary Data 4 [file 41467_2022_35337_MOESM6_ESM.docx]

| **Author (Year)** | **1** | **2** | **3** | **4** | **5** | **6** | **7** | **8** | **9** | **10** | **11** | **12** | **13** | **14** | **15** | **16** | **Review Quality** |
| --- | --- | --- | --- | --- | --- | --- | --- | --- | --- | --- | --- | --- | --- | --- | --- | --- | --- |
| Bensi et al. (2020) ^1^ | Y | PY | N | PY | Y | N | N | PY | Y/Y | N | Y/Y | N | N | Y | NA | Y | Critically Low |
| Wagle et al. (2018) ^2^ | Y | Y | Y | N | Y | N | N | Y | Y/Y | N | Y/Y | N | N | Y | NA | Y | Critically Low |
| Yang et al. (2018) ^3^ | Y | N | N | PY | Y | Y | N | PY | Y/Y | N | Y/Y | N | N | Y | NA | Y | Critically Low |
| Coelho et al. (2020) ^4^ | Y | PY | N | N | Y | N | N | N | Y/Y | N | Y/Y | N | N | Y | N | Y | Critically Low |
| de Lima et al. (2020) ^5^ | Y | PY | N | N | Y | Y | Y | N | Y/Y | N | Y/Y | N | N | N | NA | Y | Critically Low |
| Kisely et al. (2015) ^6^ | Y | PY | N | PY | Y | Y | N | Y | Y/Y | N | Y/Y | N | N | Y | Y | Y | Critically Low |
| Liu T et al. (2021) ^7^ | Y | PY | Y | PY | Y | Y | N | Y | Y/Y | N | Y/Y | N | N | Y | Y | Y | Critically Low |
| Skeie et al. (2019) ^8^ | Y | PY | Y | N | Y | Y | N | Y | Y/Y | N | Y/Y | N | Y | Y | Y | Y | Critically Low |
| Cademartori et al. (2018) ^9^ | Y | Y | Y | PY | N | Y | Y | PY | Y/Y | N | Y/Y | N | N | Y | NA | Y | Low |
| Zhou et al. (2017) ^10^ | Y | PY | N | N | Y | Y | N | PY | Y/Y | N | Y/Y | N | N | N | Y | Y | Critically Low |
| Didilescu et al. (2020) ^11^ | Y | PY | N | N | Y | N | N | PY | Y/Y | N | Y/Y | N | N | N | NA | N | Critically Low |
| Mahajan et al. (2021) ^12^ | Y | N | Y | N | Y | Y | N | PY | Y/Y | N | Y/Y | N | N | Y | Y | Y | Critically Low |
| Beukers et al. (2021) ^13^ | Y | Y | Y | N | Y | N | N | Y | Y/Y | N | Y/Y | N | N | Y | Y | Y | Critically Low |
| Papageorgiou et al. (2017) ^14^ | Y | Y | Y | Y | Y | Y | Y | Y | Y/Y | N | Y/Y | N | Y | Y | NA | Y | Moderate |
| Akcalı et al. (2019) ^15^ | Y | Y | N | PY | Y | Y | Y | Y | Y/Y | N | Y/Y | N | Y | Y | Y | Y | Moderate |
| Cerutti-Kopplin et al. (2016) ^16^ | Y | Y | Y | PY | Y | Y | N | Y | Y/Y | N | Y/Y | N | Y | Y | NA | Y | Low |
| Dai et al. (2015) ^17^ | Y | N | Y | N | Y | Y | Y | Y | Y/Y | N | Y/Y | N | N | Y | NA | Y | Critically Low |
| Nascimento et al. (2018) ^18^ | Y | Y | Y | PY | Y | Y | Y | Y | Y/Y | N | Y/Y | N | Y | Y | Y | Y | Moderate |
| Li et al. (2015) ^19^ | Y | N | Y | Y | Y | Y | Y | Y | Y/Y | N | Y/Y | N | N | Y | Y | Y | Critically Low |
| Galletti et al. (2019) ^20^ | Y | PY | Y | Y | Y | Y | N | Y | Y/Y | N | Y/Y | Y | Y | Y | NA | Y | Low |
| Muñoz Aguilera et al. (2020) ^21^ | Y | Y | Y | Y | Y | Y | N | Y | Y/Y | N | Y/Y | Y | Y | Y | Y | Y | Low |
| Kisely et al. (2015) ^22^ | Y | PY | N | PY | Y | N | N | Y | Y/Y | N | Y/Y | N | N | Y | Y | Y | Critically Low |
| Kapellas et al. (2019) ^23^ | Y | Y | Y | PY | Y | N | N | Y | Y/Y | N | Y/Y | Y | Y | Y | Y | Y | Low |
| Nascimento et al. (2015) ^24^ | Y | PY | Y | PY | Y | Y | Y | Y | Y/Y | N | Y/Y | N | Y | Y | NA | Y | Moderate |
| Nadim et al. (2020) ^25^ | Y | N | Y | PY | N | N | N | Y | Y/Y | N | Y/Y | N | N | N | Y | Y | Critically Low |
| Ioannidou et al. (2006) ^26^ | Y | N | Y | N | Y | N | N | Y | Y/Y | N | Y/Y | Y | Y | Y | N | N | Critically Low |
| Jerônimo et al. (2020) ^27^ | Y | PY | Y | PY | Y | N | N | Y | Y/Y | N | Y/Y | N | N | Y | N | N | Critically Low |
| Darnaud et al. (2021) ^28^ | Y | N | Y | Y | Y | N | Y | Y | Y/Y | N | Y/Y | N | N | Y | N | Y | Critically Low |
| Didilescu et al. (2021) ^29^ | Y | PY | Y | PY | N | N | N | Y | Y/Y | N | Y/Y | N | N | Y | NA | Y | Critically Low |
| Gobin et al. (2020) ^30^ | Y | Y | Y | N | Y | Y | N | Y | Y/Y | Y | Y/Y | N | N | Y | Y | Y | Critically Low |
| Wei et al. (2021) ^31^ | Y | PY | Y | PY | Y | Y | N | Y | Y/Y | N | Y/Y | Y | Y | Y | NA | Y | Low |
| Guo et al. (2021) ^32^ | Y | PY | Y | PY | N | Y | N | Y | Y/Y | N | Y/Y | N | N | Y | NA | Y | Critically Low |
| Akram et al. (2016) ^33^ | Y | PY | Y | PY | Y | Y | Y | Y | Y/Y | N | Y/Y | N | N | Y | N | Y | Critically Low |
| Zhu et al. (2017) ^34^ | Y | Y | Y | PY | Y | Y | N | N | Y/Y | N | Y/Y | N | Y | Y | Y | Y | Low |
| Qi et al. (2021) ^35^ | Y | PY | Y | N | N | Y | N | Y | Y/Y | N | Y/Y | Y | Y | Y | Y | Y | Low |
| Jordão et al. (2020) ^36^ | Y | N | Y | N | Y | N | N | Y | Y/Y | N | Y/Y | N | N | Y | Y | Y | Critically Low |
| Teshome et al. (2016) ^37^ | Y | PY | Y | PY | Y | Y | N | Y | Y/Y | N | Y/Y | N | Y | Y | N | Y | Low |
| Simpson et al. (2015) ^38^ | Y | Y | Y | Y | Y | Y | Y | Y | Y/Y | Y | Y/Y | Y | Y | Y | Y | Y | High |
| Iheozor-Ejiofor et al. (2017) ^39^ | Y | PY | Y | PY | Y | Y | Y | Y | Y/Y | Y | Y/Y | Y | Y | Y | Y | Y | High |
| Galdino et al. (2021) ^40^ | Y | Y | Y | PY | Y | Y | Y | Y | Y/Y | N | Y/Y | N | N | Y | Y | Y | Low |
| Chen et al. (2015) ^41^ | Y | Y | Y | PY | Y | N | N | Y | Y/Y | N | Y/Y | N | N | Y | Y | Y | Critically Low |
| Ali et al. (2021) ^42^ | Y | Y | Y | PY | Y | N | N | Y | Y/Y | N | Y/Y | Y | Y | Y | NA | Y | Low |
| Nascimento et al. (2016) ^43^ | Y | PY | Y | N | Y | Y | Y | Y | Y/Y | N | Y/Y | N | Y | Y | N | Y | Critically Low |
| Yue et al. (2020) ^44^ | Y | PY | Y | N | Y | Y | N | PY | Y/Y | N | Y/Y | N | Y | Y | Y | Y | Critically Low |
| Wang et al. (2020) ^45^ | Y | PY | Y | N | Y | Y | Y | Y | Y/Y | N | Y/Y | Y | Y | Y | Y | Y | Low |
| Otero Rey et al. (2019) ^46^ | Y | PY | Y | N | Y | Y | N | Y | Y/Y | N | Y/Y | N | N | Y | N | Y | Critically Low |
| Dioguardi et al. (2019) ^47^ | Y | N | Y | N | Y | Y | N | Y | Y/Y | N | Y/Y | N | N | Y | NA | Y | Critically Low |
| Cao et al. (2019) ^48^ | Y | Y | Y | PY | Y | Y | Y | Y | Y/Y | N | Y/Y | N | N | Y | Y | Y | Low |
| Corbella et al. (2013) ^49^ | Y | N | Y | PY | N | N | N | Y | Y/Y | N | Y/Y | N | N | Y | N | Y | Critically Low |
| Wang et al. (2016) ^50^ | Y | Y | Y | Y | Y | Y | Y | Y | Y/Y | Y | Y/Y | Y | Y | Y | NA | Y | High |
| Gopinath et al. (2020) ^51^ | Y | Y | Y | N | Y | Y | N | Y | Y/Y | N | Y/Y | N | N | N | Y | Y | Critically Low |
| Rapone et al. (2020) ^52^ | Y | N | Y | N | Y | Y | N | Y | N/N | N | Y/Y | N | Y | N | N | Y | Critically Low |
| Wu et al. (2020) ^53^ | Y | Y | Y | PY | N | Y | N | Y | Y/Y | N | Y/Y | N | N | Y | Y | Y | Critically Low |
| Botero et al. (2020) ^54^ | Y | Y | N | N | Y | Y | Y | Y | Y/Y | N | Y/Y | Y | Y | Y | Y | Y | Low |
| Zhang et al. (2022) ^55^ | Y | PY | Y | PY | Y | Y | N | N | Y/Y | N | Y/Y | N | N | Y | N | Y | Critically Low |
| Schmitt et al. (2015) ^56^ | Y | PY | Y | N | Y | N | Y | Y | Y/Y | N | Y/Y | Y | Y | Y | NA | Y | Low |
| Atieh et al. (2014) ^57^ | Y | PY | Y | N | N | N | N | Y | Y/Y | N | Y/Y | N | N | Y | N | Y | Critically Low |
| Corbella et al. (2016) ^58^ | Y | N | Y | PY | Y | Y | Y | Y | Y/Y | N | Y/Y | Y | Y | Y | N | N | Critically Low |
| Hua et al. (2016) ^59^ | Y | Y | Y | PY | Y | Y | Y | Y | Y/Y | Y | Y/Y | Y | Y | Y | Y | Y | High |
| Araújo et al. (2016) ^60^ | Y | PY | Y | N | Y | Y | N | Y | Y/Y | N | Y/Y | N | N | Y | N | Y | Critically Low |
| Ziukaite et al. (2018) ^61^ | Y | Y | Y | N | Y | Y | N | Y | Y/Y | N | Y/Y | N | N | Y | Y | Y | Critically Low |
| Ferreira et al. (2019) ^62^ | Y | Y | Y | Y | N | N | Y | Y | Y/Y | N | Y/Y | N | N | N | NA | Y | Low |
| Teeuw et al. (2014) ^63^ | Y | Y | Y | PY | Y | Y | Y | Y | Y/Y | N | Y/Y | N | N | Y | NA | Y | Low |
| Papageorgiou et al. (2015) ^64^ | Y | Y | Y | Y | Y | Y | Y | Y | Y/Y | N | Y/Y | N | Y | Y | NA | Y | Moderate |
| Tomás et al. (2012) ^65^ | Y | PY | Y | PY | Y | N | N | Y | Y/Y | N | Y/Y | N | N | Y | NA | Y | Critically Low |
| Peng et al. (2019) ^66^ | Y | PY | Y | PY | Y | Y | Y | Y | Y/Y | N | Y/Y | Y | N | Y | Y | Y | Low |
| Ferreira et al. (2019) ^67^ | Y | Y | Y | Y | Y | Y | Y | Y | Y/Y | N | Y/Y | Y | Y | Y | N | Y | Low |
| da Silva et al. (2021) ^68^ | Y | Y | Y | N | Y | Y | N | Y | Y/Y | N | Y/Y | N | N | Y | N | Y | Critically Low |
| Daudt et al. (2018) ^69^ | Y | Y | Y | PY | Y | Y | N | Y | Y/Y | N | Y/Y | N | N | Y | Y | Y | Critically Low |
| Zhao et al. (2018) ^70^ | Y | N | Y | N | Y | N | N | PY | Y/Y | N | Y/Y | N | Y | Y | Y | Y | Critically Low |
| Moraschini et al. (2016) ^71^ | Y | PY | Y | Y | Y | N | N | Y | Y/Y | N | Y/Y | N | Y | N | Y | Y | Low |
| Leira et al. (2017) ^72^ | Y | Y | Y | PY | Y | Y | N | Y | Y/Y | N | Y/Y | Y | Y | Y | NA | Y | Low |
| Moraschini et al. (2018) ^73^ | Y | PY | Y | Y | Y | Y | N | Y | Y/Y | N | Y/Y | N | Y | Y | N | Y | Critically Low |
| Joshi et al. (2019) ^74^ | Y | PY | N | N | Y | Y | N | Y | Y/Y | N | Y/Y | N | N | Y | N | N | Critically Low |
| Conde-Agudelo et al. (2008) ^75^ | Y | PY | Y | PY | N | N | N | Y | N/N | N | Y/Y | Y | Y | Y | Y | Y | Critically Low |
| Simpson et al. (2010) ^76^ | Y | Y | Y | Y | Y | Y | Y | Y | Y/Y | Y | Y/Y | Y | Y | Y | Y | Y | High |
| Boutin et al. (2013) ^77^ | Y | PY | Y | PY | Y | Y | N | Y | Y/Y | N | Y/Y | Y | Y | Y | N | Y | Critically Low |
| Suvan et al. (2011) ^78^ | Y | Y | Y | Y | Y | Y | N | Y | Y/Y | N | Y/Y | N | N | N | Y | Y | Critically Low |
| Moura-Grec et al. (2014) ^79^ | Y | N | Y | N | Y | Y | N | Y | N/N | N | Y/Y | N | Y | Y | N | N | Critically Low |
| Machado et al. (2020) ^80^ | Y | Y | Y | Y | Y | Y | N | Y | Y/Y | N | Y/Y | Y | Y | Y | NA | Y | Low |
| de Oliveira Ferreira et al. (2019) ^81^ | Y | N | Y | Y | Y | N | Y | Y | Y/Y | N | Y/Y | N | N | Y | NA | Y | Critically Low |
| Monje et al. (2017) ^82^ | Y | PY | Y | Y | Y | Y | Y | Y | Y/Y | N | Y/Y | Y | Y | Y | NA | Y | High |
| Martens et al. (2017) ^83^ | Y | Y | Y | Y | Y | Y | N | Y | Y/Y | N | Y/Y | N | N | Y | Y | Y | Critically Low |
| Esteves Lima et al. (2016) ^84^ | Y | Y | Y | PY | Y | Y | Y | Y | Y/Y | N | Y/Y | N | Y | Y | NA | Y | Critically Low |
| Li et al. (2021) ^85^ | Y | PY | Y | PY | N | N | N | Y | Y/Y | N | Y/Y | N | N | Y | Y | Y | Critically Low |
| Rosa et al. (2012) ^86^ | Y | N | Y | PY | Y | Y | N | Y | Y/Y | N | Y/Y | N | N | Y | Y | N | Critically Low |
| Leira et al. (2017) ^87^ | Y | Y | Y | PY | Y | Y | N | Y | Y/Y | N | Y/Y | N | N | Y | NA | Y | Critically Low |
| Hussain et al. (2020) ^88^ | Y | Y | Y | Y | Y | Y | Y | Y | Y/Y | N | Y/Y | Y | Y | Y | NA | Y | High |
| Botelho et al. (2018) ^89^ | Y | PY | Y | N | Y | Y | N | Y | Y/Y | N | Y/Y | N | Y | Y | Y | Y | Critically Low |
| Zheng et al. (2021) ^90^ | Y | Y | Y | N | Y | N | N | PY | Y/Y | N | Y/Y | Y | Y | Y | Y | Y | Critically Low |
| Chambrone et al. (2011) ^91^ | Y | Y | Y | Y | Y | Y | Y | Y | Y/Y | Y | Y/Y | N | Y | Y | NA | Y | High |
| Jain A et al. (2019) ^92^ | Y | N | Y | N | Y | Y | N | Y | Y/Y | N | Y/Y | N | Y | N | Y | Y | Critically Low |
| Martin-Cabezas et al. (2016) ^93^ | Y | N | Y | N | N | N | N | Y | Y/Y | N | Y/Y | N | N | Y | N | Y | Critically Low |
| Shang & Gao (2021) ^94^ | Y | Y | Y | PY | Y | N | N | Y | N/N | N | Y/Y | Y | Y | Y | Y | N | Critically Low |
| Chambrone et al. (2013) ^95^ | Y | Y | Y | PY | Y | Y | Y | Y | Y/Y | N | Y/Y | Y | Y | Y | NA | Y | High |
| Chambrone et al. (2011) ^96^ | Y | PY | Y | Y | Y | Y | Y | Y | Y/Y | Y | Y/Y | N | Y | Y | NA | Y | High |
| Paraskevas et al. (2008) ^97^ | Y | N | Y | N | Y | N | Y | Y | Y/Y | N | Y/Y | N | N | N | N | Y | Critically Low |
| Fagundes et al. (2019) ^98^ | Y | PY | Y | PY | Y | Y | N | Y | Y/Y | N | Y/Y | N | N | Y | NA | Y | Critically Low |
| da Silva et al. (2017) ^99^ | Y | PY | Y | PY | Y | Y | Y | Y | Y/Y | N | Y/Y | N | N | Y | N | Y | Critically Low |
| Bi et al. (2019) ^100^ | Y | N | Y | PY | Y | Y | N | Y | Y/Y | N | Y/Y | N | N | Y | Y | Y | Critically Low |
| Cabanillas-Balsera et al. (2019) ^101^ | Y | PY | Y | PY | Y | N | N | N | Y/Y | N | Y/Y | N | N | N | N | Y | Critically Low |
| Nibali et al. (2013) ^102^ | Y | Y | Y | PY | Y | Y | N | Y | Y/Y | N | Y/Y | Y | N | Y | Y | Y | Critically Low |
| Gomes-Filho et al. (2020) ^103^ | Y | PY | Y | PY | Y | Y | N | Y | Y/Y | N | Y/Y | N | N | Y | Y | Y | Critically Low |
| Kim et al. (2012) ^104^ | Y | PY | Y | PY | Y | Y | N | Y | Y/Y | N | Y/Y | N | N | Y | Y | Y | Low |
| Maldonado et al. (2018) ^105^ | Y | N | Y | N | Y | N | N | Y | Y/Y | N | Y/Y | N | N | Y | N | Y | Critically Low |
| Corbella et al. (2018) ^106^ | Y | Y | Y | PY | Y | Y | Y | Y | Y/Y | N | Y/Y | Y | Y | Y | N | Y | Low |
| Chrcanovic et al. (2014) ^107^ | Y | Y | Y | PY | Y | Y | N | Y | Y/Y | N | Y/Y | N | N | Y | Y | Y | Critically Low |
| Tang et al. (2017) ^108^ | Y | PY | Y | PY | Y | Y | N | Y | Y/Y | Y | Y/Y | N | N | Y | Y | Y | Critically Low |
| Deng et al. (2013) ^109^ | Y | N | Y | PY | Y | Y | N | Y | Y/Y | N | Y/Y | N | N | Y | N | N | Critically Low |
| Gupta et al. (2020) ^110^ | Y | PY | Y | N | Y | Y | Y | Y | Y/Y | N | Y/Y | N | N | Y | Y | Y | Critically Low |
| Kaur et al. (2014) ^111^ | Y | N | Y | PY | Y | N | N | Y | Y/Y | N | Y/Y | N | N | N | N | Y | Critically Low |
| Gomes et al. (2013) ^112^ | Y | PY | Y | PY | Y | N | N | Y | Y/Y | N | Y/Y | N | N | Y | N | Y | Critically Low |
| Wang et al. (2021) ^113^ | Y | PY | Y | PY | Y | Y | N | Y | Y/Y | N | Y/Y | Y | Y | Y | Y | Y | Low |
| Esteves Lima et al. (2021) ^114^ | Y | PY | Y | PY | Y | N | Y | Y | Y/Y | N | Y/Y | N | N | N | N | Y | Critically Low |
| Abariga & Whitcomb (2016) ^115^ | Y | N | Y | N | N | Y | N | Y | Y/Y | N | Y/Y | Y | Y | Y | Y | Y | Critically Low |
| Engebretson & Kocher (2013) ^116^ | Y | PY | Y | N | N | N | N | Y | Y/Y | N | Y/Y | N | N | Y | Y | Y | Critically Low |
| Figuero et al. (2013) ^117^ | Y | PY | Y | N | Y | Y | N | Y | Y/Y | Y | Y/Y | N | N | Y | Y | Y | Critically Low |
| Kunnen et al. (2010) ^118^ | Y | N | Y | N | Y | N | Y | Y | Y/Y | N | Y/Y | Y | Y | Y | N | Y | Critically Low |
| Machado et al. (2020) ^119^ | Y | PY | Y | Y | Y | Y | N | Y | Y/Y | N | Y/Y | Y | Y | Y | NA | Y | High |
| Georgiou et al. (2019) ^120^ | Y | PY | Y | PY | Y | N | Y | Y | Y/Y | Y | Y/Y | N | N | Y | N | Y | Critically Low |
| Garde et al. (2019) ^121^ | Y | Y | Y | PY | Y | N | N | Y | Y/Y | N | Y/Y | N | N | Y | N | Y | Critically Low |
| Lorenzo-Pouso et al. (2021) ^122^ | Y | Y | Y | PY | Y | Y | N | Y | Y/Y | N | Y/Y | Y | Y | Y | Y | Y | Low |
| Romandini et al. (2021) ^123^ | Y | Y | Y | PY | Y | Y | Y | Y | Y/Y | N | Y/Y | N | N | Y | Y | Y | Low |
| Artese et al. (2015) ^124^ | Y | Y | Y | N | Y | Y | N | Y | Y/Y | N | Y/Y | N | N | Y | NA | Y | Critically Low |
| Segura-Egea et al. (2016) ^125^ | Y | Y | Y | PY | Y | N | Y | Y | Y/Y | N | Y/Y | N | N | Y | N | Y | Critically Low |
| Chen et al. (2021)^126^ | Y | PY | Y | PY | Y | Y | N | Y | Y/Y | N | Y/Y | Y | Y | Y | Y | Y | Low |
| Maisonneuve et al. (2017) ^127^ | Y | N | Y | PY | Y | Y | N | Y | N/N | N | Y/Y | N | N | Y | Y | Y | Critically Low |
| Bouziane et al. (2012) ^128^ | Y | PY | Y | Y | Y | Y | N | Y | Y/Y | N | Y/Y | N | N | Y | NA | Y | Critically Low |
| Darré et al. (2008) ^129^ | Y | N | Y | Y | Y | N | N | Y | Y/Y | N | Y/Y | N | N | Y | Y | Y | Critically Low |
| Machado et al. (2021) ^130^ | Y | Y | Y | Y | Y | Y | Y | Y | Y/Y | Y | Y/Y | Y | Y | Y | Y | Y | High |
| Wu et al. (2020) ^131^ | Y | Y | Y | PY | N | N | N | Y | Y/Y | N | Y/Y | N | N | Y | N | Y | Critically Low |
| Lü et al. (2011) ^132^ | Y | N | Y | N | Y | N | N | Y | Y/Y | N | Y/Y | N | Y | Y | N | N | Critically Low |
| Corbella et al. (2012) ^133^ | Y | N | Y | N | Y | N | N | Y | N/N | N | Y/Y | N | N | N | Y | N | Critically Low |
| Wijarnpreecha et al. (2020) ^134^ | Y | PY | Y | PY | Y | Y | N | Y | Y/Y | N | Y/Y | N | N | Y | Y | Y | Critically Low |
| Lorenzo-Pouso et al. (2020) ^135^ | Y | PY | Y | N | Y | Y | N | Y | Y/Y | N | Y/Y | N | Y | Y | N | Y | Critically Low |
| Demmer et al. (2013) ^136^ | Y | PY | Y | Y | Y | Y | N | Y | Y/Y | N | Y/Y | N | N | Y | Y | Y | Critically Low |
| Polyzos et al. (2010) ^137^ | Y | PY | Y | N | N | Y | N | Y | Y/Y | N | Y/Y | N | Y | N | Y | Y | Critically Low |
| Martorano-Fernandes et al. (2020) ^138^ | Y | PY | Y | Y | N | Y | N | Y | Y/Y | N | Y/Y | Y | Y | Y | N | Y | Critically Low |
| Peña et al. (2021) ^139^ | Y | PY | Y | N | N | N | Y | Y | Y/Y | N | Y/Y | N | N | N | Y | Y | Critically Low |
| Botelho et al. (2021) ^140^ | Y | Y | Y | Y | Y | Y | Y | Y | Y/Y | Y | Y/Y | Y | Y | Y | Y | Y | High |
| Roca-Millan et al. (2018) ^141^ | Y | N | N | N | N | N | N | Y | Y/Y | N | Y/Y | N | N | Y | NA | Y | Critically Low |
| Jensen et al. (2021) ^142^ | Y | PY | N | N | Y | N | N | Y | Y/Y | N | Y/Y | N | Y | N | Y | Y | Critically Low |
| Manrique-Corredor et al. (2019) ^143^ | Y | N | Y | N | Y | Y | N | Y | Y/Y | N | Y/Y | N | Y | Y | Y | Y | Critically Low |
| Ungprasert et al. (2017) ^144^ | Y | PY | Y | N | Y | Y | N | Y | Y/Y | N | Y/Y | N | N | Y | Y | Y | Critically Low |
| Liu et al. (2017) ^145^ | Y | Y | Y | PY | Y | N | N | Y | N/N | N | Y/Y | Y | Y | N | N | Y | Critically Low |
| Teeuw et al. (2010) ^146^ | Y | PY | Y | N | N | N | Y | Y | Y/Y | N | Y/Y | N | N | N | NA | Y | Critically Low |
| Farook et al. (2021) ^147^ | Y | Y | Y | PY | Y | Y | N | Y | Y/Y | N | Y/Y | N | Y | Y | NA | Y | Low |
| Wang et al. (2014) ^148^ | Y | N | Y | PY | Y | Y | N | Y | Y/Y | N | Y/Y | N | N | Y | Y | Y | Critically Low |
| Hu et al. (2021) ^149^ | Y | Y | Y | Y | Y | Y | N | Y | Y/Y | N | Y/Y | N | N | Y | NA | Y | Critically Low |
| Schwartz et al. (2018) ^150^ | Y | PY | Y | PY | Y | Y | N | Y | Y/Y | N | Y/Y | Y | Y | Y | N | Y | Critically Low |
| Bahekar et al. (2007) ^151^ | Y | N | Y | PY | Y | Y | N | Y | Y/Y | N | Y/Y | Y | Y | N | N | N | Critically Low |
| Hsu et al. (2019) ^152^ | Y | Y | Y | PY | Y | Y | Y | Y | Y/Y | N | Y/Y | N | N | Y | Y | Y | Low |
| Fuggle et al. (2016) ^153^ | Y | PY | Y | N | Y | Y | N | Y | Y/Y | N | Y/Y | N | N | Y | N | Y | Critically Low |
| Stöhr et al. (2021) ^154^ | Y | Y | Y | PY | Y | Y | Y | Y | Y/Y | N | Y/Y | N | N | Y | Y | Y | Low |
| Ratz et al. (2015) ^155^ | Y | PY | N | PY | Y | N | N | Y | Y/Y | Y | Y/Y | N | N | Y | NA | Y | Critically Low |
| Wu et al. (2020) ^156^ | Y | N | Y | PY | N | N | Y | Y | Y/Y | N | Y/Y | N | N | Y | Y | Y | Critically Low |
| Al-Jewair et al. (2015) ^157^ | Y | Y | Y | Y | Y | Y | N | Y | Y/Y | N | Y/Y | N | N | N | NA | Y | Critically Low |
| Chaffee & Weston (2010) ^158^ | Y | PY | Y | PY | Y | Y | N | Y | Y/Y | N | Y/Y | Y | Y | Y | Y | Y | Low |
| da Silva et al. (2021) ^159^ | Y | PY | Y | PY | Y | N | Y | Y | Y/Y | N | Y/Y | N | N | N | Y | Y | Low |
| Larvin et al. (2021) ^160^ | Y | PY | Y | N | N | N | N | Y | Y/Y | N | Y/Y | N | N | Y | Y | Y | Critically Low |
| Nepomuceno et al. (2017) ^161^ | Y | PY | Y | N | N | Y | N | Y | Y/Y | N | Y/Y | N | Y | Y | Y | Y | Critically Low |
| Qiao et al. (2020) ^162^ | Y | N | Y | PY | Y | Y | N | Y | Y/Y | N | Y/Y | N | N | Y | Y | Y | Critically Low |
| Dicembrini et al. (2020) ^163^ | Y | PY | Y | N | Y | Y | N | Y | Y/Y | N | Y/Y | N | N | N | Y | Y | Critically Low |
| Xiao et al. (2020) ^164^ | Y | Y | N | PY | Y | Y | N | Y | Y/Y | N | Y/Y | N | N | Y | Y | Y | Critically Low |
| Yang et al. (2018) ^165^ | Y | PY | N | N | Y | Y | N | Y | Y/Y | N | Y/Y | N | N | N | N | Y | Critically Low |
| Orlandi et al. (2014) ^166^ | Y | Y | Y | Y | Y | Y | N | Y | Y/Y | N | Y/Y | Y | Y | Y | Y | Y | Low |
| Zhang Y et al. (2021) ^167^ | Y | PY | Y | N | N | Y | Y | Y | Y/Y | N | Y/Y | N | N | Y | N | Y | Critically Low |
| Zhang J et al. (2021) ^168^ | Y | Y | Y | PY | Y | Y | N | Y | Y/Y | N | Y/Y | N | N | Y | N | Y | Critically Low |
| Moliner-Sánchez et al. (2020) ^169^ | Y | PY | Y | PY | Y | Y | N | Y | Y/Y | N | Y/Y | N | N | Y | Y | Y | Critically Low |
| Baeza et al. (2020) ^170^ | Y | Y | Y | N | Y | Y | Y | Y | Y/Y | N | Y/Y | N | N | Y | Y | Y | Critically Low |
| Rutter-Locher et al. (2017) ^171^ | Y | PY | Y | N | Y | Y | N | Y | Y/Y | N | Y/Y | N | N | Y | N | Y | Critically Low |
| Chen et al. (2020) ^172^ | Y | PY | Y | PY | N | Y | N | Y | Y/Y | N | Y/Y | Y | Y | Y | Y | Y | Low |
| Koletsi et al. (2021) ^173^ | Y | PY | Y | Y | Y | Y | N | Y | Y/Y | N | Y/Y | Y | Y | Y | Y | N | Moderate |
| Ozturk et al. (2021) ^174^ | Y | Y | Y | PY | N | N | N | Y | Y/Y | N | Y/Y | N | N | Y | N | Y | Critically Low |
| Zhou et al. (2019) ^175^ | Y | Y | Y | N | N | Y | N | Y | Y/Y | N | Y/Y | N | N | Y | N | Y | Critically Low |
| Lv et al. (2020) ^176^ | Y | PY | Y | PY | Y | Y | N | Y | Y/Y | N | Y/Y | N | N | Y | Y | Y | Critically Low |
| Xu et al. (2020) ^177^ | Y | PY | Y | PY | Y | Y | N | Y | Y/Y | N | Y/Y | Y | Y | Y | N | Y | Critically Low |
| Silva et al. (2022) ^178^ | Y | PY | Y | PY | Y | Y | N | Y | Y/Y | N | Y/Y | N | Y | N | N | Y | Critically Low |
| Calderaro et al. (2016) ^179^ | Y | N | N | PY | Y | Y | N | N | Y/Y | N | Y/Y | N | N | Y | N | Y | Critically Low |
| Ren et al. (2016) ^180^ | Y | Y | Y | Y | Y | Y | Y | Y | Y/Y | N | Y/Y | Y | N | Y | NA | N | Moderate |
| Zainal Abidin et al. (2021) ^181^ | Y | Y | N | PY | N | N | N | Y | Y/Y | N | Y/Y | N | N | Y | N | Y | Critically Low |
| Hussain et al. (2021) ^182^ | Y | Y | Y | Y | Y | Y | N | Y | Y/Y | N | Y/Y | Y | N | N | NA | Y | Critically Low |
| Mirzaei et al. (2021) ^183^ | Y | Y | Y | PY | Y | N | N | Y | Y/Y | N | Y/Y | Y | Y | Y | Y | Y | Low |
| Maarse et al. (2019) ^184^ | Y | Y | Y | N | Y | N | N | Y | Y/Y | N | Y/Y | N | N | N | N | N | Critically Low |
| Souza et al. (2019) ^185^ | Y | Y | Y | PY | Y | Y | Y | Y | Y/Y | N | Y/Y | N | N | Y | N | Y | Critically Low |
| Jalili et al. (2020) ^186^ | Y | N | N | N | Y | Y | N | N | Y/Y | N | Y/Y | N | N | Y | N | Y | Critically Low |
| Silveira et al. (2020) ^187^ | Y | PY | N | Y | Y | Y | N | Y | Y/Y | N | Y/Y | N | Y | Y | Y | Y | Low |
| Aminoshariae et al. (2020) ^188^ | Y | PY | N | N | N | N | Y | Y | Y/Y | N | Y/Y | Y | N | Y | NA | Y | Critically Low |
| Aminoshariae et al. (2018) ^189^ | Y | PY | N | N | N | N | Y | Y | Y/Y | N | Y/Y | Y | N | Y | NA | Y | Critically Low |
| Alvarenga et al. (2019) ^190^ | Y | Y | Y | Y | Y | Y | Y | Y | Y/Y | N | Y/Y | Y | Y | Y | Y | Y | High |
| Araújo et al. (2020) ^191^ | Y | Y | Y | PY | Y | Y | N | N | Y/Y | N | Y/Y | N | N | Y | Y | Y | Critically Low |
| Hermont et al. (2014) ^192^ | Y | N | Y | Y | Y | Y | Y | Y | Y/Y | N | Y/Y | N | N | Y | NA | Y | Critically Low |
| Lockhart et al. (2019) ^193^ | Y | Y | Y | PY | Y | Y | Y | Y | Y/Y | N | Y/Y | Y | Y | Y | N | Y | Low |
| Wang et al. (2020) ^194^ | Y | N | Y | N | N | N | N | Y | N/N | N | Y/Y | N | N | Y | Y | Y | Critically Low |
| Jiang et al. (2021) ^195^ | Y | Y | N | Y | N | N | N | Y | Y/Y | N | Y/Y | N | N | Y | Y | Y | Critically Low |
| Fang et al. (2018) ^196^ | Y | PY | N | N | N | N | N | Y | Y/Y | N | Y/Y | N | N | Y | Y | Y | Critically Low |
| Souto-Souza et al. (2020) ^197^ | Y | PY | Y | PY | Y | N | N | PY | Y/Y | N | Y/Y | N | Y | Y | Y | Y | Low |
| Rios-Osorio et al. (2020) ^198^ | Y | N | Y | Y | N | Y | N | Y | Y/Y | N | Y/Y | Y | N | Y | NA | Y | Critically Low |
| Blaizot et al. (2009) ^199^ | Y | N | Y | PY | Y | Y | N | Y | Y/Y | N | Y/Y | N | N | Y | Y | N | Critically Low |
| Oh et al. (2018) ^200^ | Y | PY | N | N | Y | Y | N | Y | Y/Y | N | Y/Y | N | N | Y | Y | Y | Critically Low |
| AlOtaibi et al. (2021) ^201^ | Y | N | Y | N | N | N | N | Y | N/N | N | Y/Y | N | N | N | NA | Y | Critically Low |
| Easwaran et al. (2021) ^202^ | Y | Y | Y | Y | Y | Y | Y | Y | Y/Y | N | Y/Y | N | N | N | NA | Y | Low |
| Ji et al. (2021) ^203^ | Y | N | Y | N | N | N | N | Y | N/N | N | Y/Y | N | N | Y | NA | Y | Critically Low |
| Sun et al. (2021) ^204^ | Y | PY | Y | PY | Y | Y | N | Y | Y/Y | N | Y/Y | N | N | Y | NA | Y | Critically Low |
| Drumond et al. (2021) ^205^ | Y | PY | Y | PY | Y | Y | Y | Y | Y/Y | N | Y/Y | N | N | Y | NA | Y | Low |
| Le et al. (2021) ^206^ | Y | PY | Y | PY | Y | N | N | Y | Y/Y | N | Y/Y | N | N | N | Y | Y | Critically Low |
| Marzouk et al. (2021) ^207^ | Y | PY | N | Y | Y | Y | Y | N | Y/Y | N | Y/Y | N | Y | Y | NA | Y | Moderate |
| Del Rei Daltro Rosa et al. (2021) ^208^ | Y | PY | Y | PY | Y | N | Y | Y | Y/Y | N | Y/Y | N | N | Y | NA | Y | Low |
| Porto et al. (2021) ^209^ | Y | PY | Y | N | Y | Y | N | Y | Y/Y | Y | Y/Y | N | Y | N | N | N | Critically Low |
| Orlandi et al. (2021) ^210^ | Y | Y | Y | PY | Y | Y | Y | Y | Y/Y | Y | Y/Y | Y | Y | Y | Y | Y | High |
| Serni et al. (2021) ^211^ | Y | PY | Y | PY | Y | Y | N | Y | Y/Y | N | Y/Y | N | N | Y | NA | Y | Critically Low |
| Andrade et al. (2022) ^212^ | Y | PY | Y | Y | Y | Y | N | Y | Y/Y | N | Y/Y | N | N | Y | NA | Y | Critically Low |
| Gusman et al. (2018) ^213^ | Y | PY | Y | N | Y | Y | Y | Y | Y/Y | N | Y/Y | N | N | Y | NA | Y | Critically Low |
| Pi et al. (2020) ^214^ | Y | Y | Y | PY | Y | Y | N | Y | Y/Y | N | Y/Y | N | N | Y | NA | N | Critically Low |
| Zhang et al. (2020) ^215^ | Y | Y | Y | PY | Y | Y | Y | Y | Y/Y | N | Y/Y | N | N | Y | NA | Y | Low |
| Hatipoğlu et al. (2022) ^216^ | Y | PY | Y | N | Y | Y | N | Y | Y/Y | N | Y/Y | Y | Y | Y | Y | Y | Critically Low |
| Xi et al. (2017) ^217^ | Y | PY | Y | PY | Y | Y | N | Y | Y/Y | N | Y/Y | N | N | Y | Y | Y | Critically Low |
| Sharifi et al. (2021) ^218^ | Y | PY | Y | PY | Y | Y | N | Y | Y/Y | N | Y/Y | N | N | Y | Y | Y | Critically Low |
| Granja et al. (2022) ^219^ | Y | PY | Y | PY | Y | Y | Y | Y | Y/Y | Y | Y/Y | N | N | Y | Y | Y | Low |
| Xu et al. (2019) ^220^ | Y | PY | Y | PY | Y | Y | N | Y | Y/Y | N | Y/Y | N | N | Y | Y | Y | Critically Low |
| Zeng et al. (2020) ^221^ | Y | Y | Y | N | Y | Y | Y | Y | Y/Y | N | Y/Y | N | N | Y | NA | Y | Critically Low |
| Hatipoğlu et al. (2022) ^222^ | Y | Y | Y | N | Y | Y | N | Y | Y/Y | N | Y/Y | Y | Y | Y | Y | Y | Critically Low |
| Arduino et al. (2022) ^223^ | Y | Y | Y | PY | Y | Y | Y | Y | Y/Y | N | Y/Y | Y | Y | Y | Y | N | Moderate |
| Lianhui et al. (2017) ^224^ | Y | PY | Y | PY | N | N | N | Y | Y/Y | N | Y/Y | N | N | Y | NA | Y | Critically Low |
| Shi et al. (2018) ^225^ | Y | PY | Y | N | Y | Y | N | Y | Y/Y | N | Y/Y | Y | Y | Y | NA | Y | Critically Low |
| Zeng et al. (2013) ^226^ | Y | PY | Y | PY | Y | Y | N | Y | N/N | N | Y/Y | N | Y | Y | Y | Y | Critically Low |
| Zeng et al. (2021) ^227^ | Y | Y | Y | N | Y | Y | N | Y | Y/Y | N | Y/Y | N | Y | Y | NA | Y | Critically Low |
| Zeng et al. (2016) ^228^ | Y | PY | Y | PY | Y | Y | Y | Y | N/N | N | Y/Y | N | Y | Y | NA | Y | Low |
| Sgolastra et al. (2013) ^229^ | Y | PY | Y | PY | Y | Y | Y | Y | Y/Y | N | Y/Y | Y | Y | Y | Y | Y | High |
| Uppal et al. (2010) ^230^ | Y | Y | Y | N | Y | N | N | Y | Y/Y | N | Y/Y | Y | Y | Y | N | N | Critically Low |
| Huang et al. (2014) ^231^ | Y | Y | Y | N | N | Y | Y | Y | Y/Y | N | Y/Y | Y | Y | Y | Y | Y | Low |
| Liew et al. (2013) ^232^ | Y | Y | Y | N | Y | Y | N | Y | Y/Y | N | Y/Y | N | Y | Y | Y | N | Critically Low |
| Xuan et al. (2021) ^233^ | Y | Y | Y | PY | Y | Y | N | Y | Y/Y | N | Y/Y | Y | Y | Y | Y | Y | Low |
| Ren et al. (2016) ^234^ | Y | N | Y | PY | N | N | N | Y | N/N | N | Y/Y | N | N | N | NA | Y | Critically Low |
| Xu et al. (2019) ^235^ | Y | PY | Y | N | Y | Y | N | Y | Y/Y | N | Y/Y | N | N | Y | Y | Y | Critically Low |
| Sun et al. (2014) ^236^ | Y | Y | Y | N | Y | Y | N | Y | Y/Y | N | Y/Y | N | Y | Y | NA | Y | Critically Low |
| George et al. (2011) ^237^ | Y | PY | Y | N | Y | Y | Y | Y | Y/Y | N | Y/Y | Y | Y | Y | N | N | Critically Low |
| Michaud et al. (2017) ^238^ | Y | N | Y | N | N | N | N | Y | N/N | N | Y/Y | N | N | Y | NA | Y | Critically Low |
| Zhong et al. (2020) ^239^ | Y | Y | Y | N | Y | N | N | Y | Y/Y | N | Y/Y | N | Y | Y | NA | Y | Critically Low |
| Le et al. (2022) ^240^ | Y | N | Y | PY | Y | Y | N | Y | Y/Y | N | Y/Y | N | N | Y | Y | Y | Critically Low |
| Noites et al. (2022) ^241^ | Y | PY | Y | N | N | N | Y | Y | Y/Y | N | Y/Y | Y | N | Y | NA | Y | Critically Low |
| Ahmadinia et al. (2022) ^242^ | Y | Y | Y | PY | Y | N | Y | Y | Y/Y | N | Y/Y | Y | Y | Y | Y | Y | Moderate |
| Sgolastra et al. (2013) ^243^ | Y | Y | Y | Y | Y | Y | Y | Y | Y/Y | N | Y/Y | Y | Y | Y | Y | Y | High |
| Ye et al. (2016) ^244^ | Y | N | Y | N | Y | Y | N | Y | N/N | N | Y/Y | N | N | N | Y | Y | Critically Low |
| Polyzos et al. (2009) ^245^ | Y | N | Y | PY | Y | Y | N | Y | N/N | N | Y/Y | N | N | Y | NA | N | Critically Low |
| Wei BJ et al. (2013) ^246^ | Y | N | Y | PY | Y | Y | Y | Y | N/N | N | Y/Y | Y | Y | Y | Y | Y | Critically Low |
| Yao et al. (2014) ^247^ | Y | Y | Y | PY | Y | Y | N | Y | N/N | N | Y/Y | N | Y | Y | Y | N | Critically Low |
| Zhu et al. (2015) ^248^ | Y | PY | Y | PY | Y | Y | N | Y | N/N | N | Y/Y | N | Y | Y | Y | Y | Critically Low |
| Ma et al. (2018) ^249^ | Y | PY | Y | PY | N | Y | N | Y | Y/Y | N | Y/Y | Y | Y | Y | NA | Y | Low |
| Li et al. (2017) ^250^ | Y | Y | Y | PY | Y | Y | N | Y | Y/Y | N | Y/Y | N | Y | Y | Y | Y | Low |
| Qin et al. (2021) ^251^ | Y | Y | Y | N | Y | Y | N | Y | Y/Y | N | Y/Y | Y | Y | Y | Y | Y | Critically Low |
| Zeng et al. (2012) ^252^ | Y | PY | Y | PY | Y | Y | N | Y | N/N | N | Y/Y | N | Y | Y | Y | Y | Critically Low |
| Zhang et al. (2020) ^253^ | Y | Y | Y | N | Y | Y | N | Y | Y/Y | N | Y/Y | Y | Y | Y | Y | Y | Critically Low |
| Wang et al. (2014) ^254^ | Y | Y | Y | PY | Y | Y | Y | Y | Y/Y | N | Y/Y | N | Y | Y | NA | Y | Moderate |
| Liu et al. (2018) ^255^ | Y | Y | Y | N | Y | Y | N | Y | Y/Y | N | Y/Y | N | Y | Y | NA | Y | Critically Low |
| Rodrigues et al. (2022) ^256^ | Y | Y | Y | N | Y | Y | Y | Y | Y/Y | N | Y/Y | N | N | Y | N | Y | Critically Low |
| Chen et al. (2019) ^257^ | Y | PY | Y | N | Y | Y | N | Y | N/N | Y | Y/Y | N | N | Y | N | Y | Critically Low |
| Liu et al. (2014) ^258^ | Y | PY | Y | N | Y | N | N | Y | Y/Y | N | Y/Y | N | N | Y | N | Y | Critically Low |
| She et al. (2020) ^259^ | Y | PY | Y | N | Y | Y | N | Y | Y/Y | N | Y/Y | N | N | Y | Y | Y | Critically Low |
| Zeng et al. (2016) ^260^ | Y | N | Y | N | Y | Y | N | Y | N/N | N | Y/Y | N | N | Y | Y | Y | Critically Low |
| Gao (2017) ^261^ | Y | N | Y | N | N | Y | N | Y | N/N | N | Y/Y | N | N | Y | Y | Y | Critically Low |
| Khodadadi et al. (2022) ^262^ | Y | PY | Y | PY | Y | N | N | Y | Y/Y | N | Y/Y | N | Y | Y | Y | Y | Low |
| Foratori-Junior et al. (2022) ^263^ | Y | PY | Y | Y | Y | Y | N | Y | Y/Y | N | Y/Y | N | N | Y | Y | Y | Critically Low |
| Zhang et al. (2021) ^264^ | Y | PY | Y | PY | Y | Y | N | Y | Y/Y | N | Y/Y | N | Y | Y | N | Y | Critically Low |
| Ma et al. (2020) ^265^ | Y | PY | Y | PY | Y | Y | N | Y | Y/Y | N | Y/Y | N | N | Y | NA | Y | Critically Low |
| Sun et al. (2021) ^266^ | Y | Y | Y | PY | N | Y | Y | Y | Y/Y | N | Y/Y | N | N | Y | Y | Y | Low |
| Li et al. (2015) ^267^ | Y | PY | Y | N | Y | Y | N | Y | Y/Y | N | Y/Y | N | N | Y | Y | Y | Critically Low |
| López-Valverde et al. (2022) ^268^ | Y | PY | Y | N | Y | N | N | Y | Y/Y | Y | Y/Y | N | N | Y | Y | N | Critically Low |
| Wei X et al. (2019) ^269^ | Y | PY | Y | N | Y | Y | N | Y | Y/Y | N | Y/Y | N | N | Y | Y | Y | Critically Low |
| Gao et al. (2021) ^270^ | Y | PY | Y | PY | N | Y | N | Y | Y/Y | N | Y/Y | N | N | Y | Y | Y | Critically Low |
| Zheng et al. (2021) ^271^ | Y | Y | Y | N | Y | Y | N | Y | Y/Y | N | Y/Y | Y | Y | Y | Y | Y | Critically Low |
| Guo et al. (2021) ^272^ | Y | Y | Y | N | Y | N | Y | Y | Y/Y | N | Y/Y | Y | Y | Y | Y | Y | Low |
| Qiao et al. (2019) ^273^ | Y | Y | Y | PY | Y | Y | N | Y | Y/Y | N | Y/Y | N | N | Y | Y | Y | Critically Low |
| Huang et al. (2021) ^274^ | Y | Y | Y | PY | Y | Y | Y | Y | Y/Y | N | Y/Y | N | N | Y | Y | Y | Low |
| Xu et al. (2021) ^275^ | Y | Y | Y | N | N | N | N | Y | Y/Y | N | Y/Y | Y | Y | Y | Y | Y | Critically Low |
| Maulani et al. (2021) ^276^ | Y | Y | Y | N | N | N | Y | Y | Y/Y | N | Y/Y | N | N | Y | Y | Y | Critically Low |
| Leng et al. (2015) ^277^ | Y | PY | Y | PY | N | N | N | Y | Y/Y | N | Y/Y | N | N | Y | Y | Y | Critically Low |
| Zhang et al. (2017) ^278^ | Y | Y | Y | N | N | Y | N | Y | Y/Y | N | Y/Y | Y | N | Y | Y | Y | Critically Low |
| Shao et al. (2018) ^279^ | Y | N | N | N | N | N | N | Y | Y/Y | N | Y/Y | N | N | N | N | N | Critically Low |
| Shi et al. (2018) ^280^ | Y | Y | Y | N | Y | Y | N | Y | Y/Y | N | Y/Y | N | N | Y | Y | Y | Critically Low |
| Qiu et al. (2020) ^281^ | Y | Y | Y | PY | Y | Y | N | Y | Y/Y | N | Y/Y | N | Y | Y | NA | Y | Low |
| Xie et al. (2018) ^282^ | Y | Y | Y | N | N | Y | Y | Y | Y/Y | N | Y/Y | N | N | Y | NA | Y | Critically Low |
| Wu et al. (2022) ^283^ | Y | Y | Y | Y | Y | Y | N | Y | Y/Y | N | Y/Y | N | N | Y | Y | Y | Critically Low |
| Chen et al. (2018) ^284^ | Y | PY | Y | PY | N | N | N | Y | N/N | N | Y/Y | N | N | Y | N | Y | Critically Low |
| Sayeed et al. (2021) ^285^ | Y | Y | Y | N | N | N | N | Y | Y/Y | N | Y/Y | N | N | Y | Y | Y | Critically Low |
| França et al. (2019) ^286^ | Y | N | Y | PY | Y | Y | N | Y | N/N | N | Y/Y | N | N | N | Y | Y | Critically Low |
| Shi et al. (2016) ^287^ | Y | PY | Y | N | Y | Y | N | Y | Y/Y | N | Y/Y | Y | Y | Y | N | Y | Critically Low |
| Wu et al. (2021) ^288^ | Y | Y | Y | PY | N | N | N | Y | Y/Y | N | Y/Y | N | N | Y | NA | Y | Critically Low |
| Simpson et al. (2022) ^289^ | Y | Y | Y | PY | Y | Y | Y | Y | Y/Y | Y | Y/Y | Y | Y | Y | Y | Y | High |
| Luo et al. (2021) ^290^ | Y | Y | Y | PY | Y | Y | Y | Y | Y/Y | Y | Y/Y | Y | Y | Y | NA | Y | High |
| Irwandi et al. 2022 ^291^ | Y | Y | Y | N | Y | N | N | Y | Y/Y | Y | Y/Y | Y | Y | Y | Y | Y | Critically Low |
| Antonarakis et al. 2013 ^292^ | Y | PY | Y | PY | Y | N | N | Y | Y/Y | N | Y/Y | N | N | N | Y | Y | Critically Low |
| Grewcock et al. 2022 ^293^ | Y | PY | Y | N | Y | Y | N | Y | Y/Y | N | Y/Y | N | Y | N | Y | Y | Critically Low |

N—No, Y—Yes, PY—Partial Yes. 1. Research questions and inclusion criteria? 2. Review methods established a priori? 3. Explanation of their selection literature search strategy? 4. Did the review authors use a comprehensive literature search strategy? 5. Study selection performed in duplicate? 6. Data selection performed in duplicate? 7. List of excluded studies and exclusions justified? 8. Description of the included studies in adequate detail? 9. Satisfactory technique for assessing the risk of bias (RoB)? 10. Report on the sources of funding for the studies included in the review? 11. If meta-analysis was performed, did the review authors use appropriate methods for statistical combination of results? 12. If meta-analysis was performed, did the review authors assess the potential impact of RoB? 13. RoB accounted when interpreting/discussing the results of the review? 14. Did the review authors provide a satisfactory explanation for, and discussion of, any heterogeneity observed in the results of the review? 15. If they performed quantitative synthesis, was publication bias performed? 16. Did the review authors report any potential sources of conflict of interest, including funding sources?.

References

1. Bensi, C., Costacurta, M. & Docimo, R. Oral health in children with cerebral palsy: A systematic review and meta-analysis. *Spec. Care Dent. Off. Publ. Am. Assoc. Hosp. Dent. Acad. Dent. Handicap. Am. Soc. Geriatr. Dent.* **40**, 401–411 (2020).

2. Wagle, M. *et al.* Dental caries and preterm birth: a systematic review and meta-analysis. *BMJ Open* **8**, e018556 (2018).

3. Yang, M. *et al.* Poor oral health in patients with schizophrenia: A systematic review and meta-analysis. *Schizophr. Res.* **201**, 3–9 (2018).

4. Coelho, A. S. *et al.* Dental caries, diabetes mellitus, metabolic control and diabetes duration: A systematic review and meta-analysis. *J. Esthet. Restor. Dent. Off. Publ. Am. Acad. Esthet. Dent. Al* **32**, 291–309 (2020).

5. de Lima, A. K. A., Amorim Dos Santos, J., Stefani, C. M., Almeida de Lima, A. de & Damé-Teixeira, N. Diabetes mellitus and poor glycemic control increase the occurrence of coronal and root caries: a systematic review and meta-analysis. *Clin. Oral Investig.* **24**, 3801–3812 (2020).

6. Kisely, S., Baghaie, H., Lalloo, R., Siskind, D. & Johnson, N. W. A systematic review and meta-analysis of the association between poor oral health and severe mental illness. *Psychosom. Med.* **77**, 83–92 (2015).

7. Liu, T., Wei, Y., Zhu, Y. & Yang, W. Caries Status and Salivary Alterations of Type-1 Diabetes Mellitus in Children and Adolescents: A Systematic Review and Meta-analysis. *J. Evid. Based Dent. Pract.* **21**, 101496 (2021).

8. Skeie, M. S. *et al.* Oral health in children and adolescents with juvenile idiopathic arthritis – a systematic review and meta-analysis. *BMC Oral Health* **19**, 285 (2019).

9. Cademartori, M. G., Gastal, M. T., Nascimento, G. G., Demarco, F. F. & Corrêa, M. B. Is depression associated with oral health outcomes in adults and elders? A systematic review and meta-analysis. *Clin. Oral Investig.* **22**, 2685–2702 (2018).

10. Zhou, N., Wong, H. M., Wen, Y. F. & Mcgrath, C. Oral health status of children and adolescents with intellectual disabilities: a systematic review and meta‐analysis. *Dev. Med. Child Neurol.* **59**, 1019–1026 (2017).

11. Didilescu, A. C., Lazu, A., Pronk, C., Vacaru, R. P. & Brand, H. S. Clinical periodontal and dental findings in liver transplant patients: a systematic review and meta-analysis. *Br. Dent. J.* **228**, 108–116 (2020).

12. Mahajan, S., Bhaskar, N., Kaur, R. K. & Jain, A. A comparison of oral health status in diabetic and non-diabetic patients receiving hemodialysis – A systematic review and meta-analysis. *Diabetes Metab. Syndr. Clin. Res. Rev.* **15**, 102256 (2021).

13. Beukers, N. G. F. M., Su, N., Loos, B. G. & van der Heijden, G. J. M. G. Lower Number of Teeth Is Related to Higher Risks for ACVD and Death—Systematic Review and Meta-Analyses of Survival Data. *Front. Cardiovasc. Med.* **8**, 621626 (2021).

14. Papageorgiou, S. N. *et al.* Inflammatory bowel disease and oral health: systematic review and a meta-analysis. *J. Clin. Periodontol.* **44**, 382–393 (2017).

15. Akcalı, A., Yıldız, M. S., Akcalı, Z., Huck, O. & Friedmann, A. Periodontal condition of patients with Thalassemia Major: A systematic review and meta-analysis. *Arch. Oral Biol.* **102**, 113–121 (2019).

16. Cerutti-Kopplin, D. *et al.* Tooth Loss Increases the Risk of Diminished Cognitive Function: A Systematic Review and Meta-analysis. *JDR Clin. Transl. Res.* **1**, 10–19 (2016).

17. Dai, R. *et al.* A systematic review and meta-analysis of clinical, microbiological, and behavioural aspects of oral health among patients with stroke. *J. Dent.* **43**, 171–180 (2015).

18. Nascimento, G. G., Leite, F. R. M., Vestergaard, P., Scheutz, F. & López, R. Does diabetes increase the risk of periodontitis? A systematic review and meta-regression analysis of longitudinal prospective studies. *Acta Diabetol.* **55**, 653–667 (2018).

19. Li, L.-W., Wong, H. M., Sun, L., Wen, Y. F. & McGrath, C. P. Anthropometric Measurements and Periodontal Diseases in Children and Adolescents: A Systematic Review and Meta-Analysis. *Adv. Nutr.* **6**, 828–841 (2015).

20. Galletti, C., Camps-Font, O., Teixido-Tura, G., Llobet-Poal, I. & Gay-Escoda, C. Association between marfan syndrome and oral health status: A systematic review and meta-analysis. *Med. Oral Patol. Oral Cirugia Bucal* 0–0 (2019) doi:10.4317/medoral.23037.

21. Muñoz Aguilera, E. *et al.* Periodontitis is associated with hypertension: a systematic review and meta-analysis. *Cardiovasc. Res.* **116**, 28–39 (2020).

22. Kisely, S., Baghaie, H., Lalloo, R. & Johnson, N. W. Association between poor oral health and eating disorders: Systematic review and meta-analysis. *Br. J. Psychiatry* **207**, 299–305 (2015).

23. Kapellas, K. *et al.* Periodontal and chronic kidney disease association: A systematic review and meta-analysis: Periodontal and chronic kidney disease. *Nephrology* **24**, 202–212 (2019).

24. Nascimento, G. G. *et al.* Is weight gain associated with the incidence of periodontitis? A systematic review and meta-analysis. *J. Clin. Periodontol.* **42**, 495–505 (2015).

25. Nadim, R. *et al.* Influence of periodontal disease on risk of dementia: a systematic literature review and a meta-analysis. *Eur. J. Epidemiol.* **35**, 821–833 (2020).

26. Ioannidou, E., Malekzadeh, T. & Dongari-Bagtzoglou, A. Effect of Periodontal Treatment on Serum C-Reactive Protein Levels: A Systematic Review and Meta-Analysis. *J. Periodontol.* **77**, 1635–1642 (2006).

27. Jerônimo, L. S., Abreu, L. G., Cunha, F. A. & Esteves Lima, R. P. Association Between Periodontitis and Nosocomial Pneumonia: A Systematic Review and Meta-analysis of Observational Studies. *Oral Health Prev. Dent.* **18**, 11–17 (2020).

28. Darnaud, C. *et al.* Association between periodontitis and pulse wave velocity: a systematic review and meta-analysis. *Clin. Oral Investig.* **25**, 393–405 (2021).

29. Didilescu, A. C. *et al.* Oral diseases after liver transplantation: a systematic review and meta-analysis. *Br. Dent. J.* **231**, 117–124 (2021).

30. Gobin, R., Tian, D., Liu, Q. & Wang, J. Periodontal Diseases and the Risk of Metabolic Syndrome: An Updated Systematic Review and Meta-Analysis. *Front. Endocrinol.* **11**, 336 (2020).

31. Wei, Y., Zhong, Y., Wang, Y. & Huang, R. Association between periodontal disease and prostate cancer: a systematic review and meta-analysis. *Med. Oral Patol. Oral Cirugia Bucal* e459–e465 (2021) doi:10.4317/medoral.24308.

32. Guo, Z. *et al.* Periodontal disease and the risk of prostate cancer: a meta-analysis of cohort studies. *Int. Braz. J. Urol.* **47**, 1120–1130 (2021).

33. Akram, Z., Abduljabbar, T., Abu Hassan, M. I., Javed, F. & Vohra, F. Cytokine Profile in Chronic Periodontitis Patients with and without Obesity: A Systematic Review and Meta-Analysis. *Dis. Markers* **2016**, 4801418 (2016).

34. Zhu, J. *et al.* Association of circulating leptin and adiponectin with periodontitis: a systematic review and meta-analysis. *BMC Oral Health* **17**, 104 (2017).

35. Qi, X., Zhu, Z., Plassman, B. L. & Wu, B. Dose-Response Meta-Analysis on Tooth Loss With the Risk of Cognitive Impairment and Dementia. *J. Am. Med. Dir. Assoc.* **22**, 2039–2045 (2021).

36. Jordão, H. W. T., Coleman, H. G., Kunzmann, A. T. & McKenna, G. The association between erosive toothwear and gastro-oesophageal reflux-related symptoms and disease: A systematic review and meta-analysis. *J. Dent.* **95**, 103284 (2020).

37. Teshome, A. & Yitayeh, A. The effect of periodontal therapy on glycemic control and fasting plasma glucose level in type 2 diabetic patients: systematic review and meta-analysis. *BMC Oral Health* **17**, 31 (2017).

38. Simpson, T. C. *et al.* Treatment of periodontal disease for glycaemic control in people with diabetes mellitus. *Cochrane Database Syst. Rev.* (2015) doi:10.1002/14651858.CD004714.pub3.

39. Iheozor-Ejiofor, Z., Middleton, P., Esposito, M. & Glenny, A.-M. Treating periodontal disease for preventing adverse birth outcomes in pregnant women. *Cochrane Database Syst. Rev.* **2017**, (2017).

40. Galdino, T. M. *et al.* Periodontal Outcomes in Liver Transplantation Individuals: A Systematic Review and Meta-analysis. *J. Gastrointestin. Liver Dis.* **30**, 122–131 (2021).

41. Chen, H., Nie, S., Zhu, Y. & Lu, M. Teeth loss, teeth brushing and esophageal carcinoma: a systematic review and meta-analysis. *Sci. Rep.* **5**, 15203 (2015).

42. Ali, A., Lassi, Z. S., Kapellas, K., Jamieson, L. & Rumbold, A. R. A systematic review and meta-analysis of the association between periodontitis and oral high-risk human papillomavirus infection. *J. Public Health* **43**, e610–e619 (2021).

43. Nascimento, G. G. *et al.* Is there a relationship between obesity and tooth loss and edentulism? A systematic review and meta-analysis: Obesity and tooth loss. *Obes. Rev.* **17**, 587–598 (2016).

44. Yue, H. *et al.* Effects of non-surgical periodontal therapy on systemic inflammation and metabolic markers in patients undergoing haemodialysis and/or peritoneal dialysis: a systematic review and meta-analysis. *BMC Oral Health* **20**, 18 (2020).

45. Wang, J. *et al.* Relationship between periodontal disease and lung cancer: A systematic review and meta‐analysis. *J. Periodontal Res.* **55**, 581–593 (2020).

46. Otero Rey, E. M., Yáñez‐Busto, A., Rosa Henriques, I. F., López‐López, J. & Blanco‐Carrión, A. Lichen planus and diabetes mellitus: Systematic review and meta‐analysis. *Oral Dis.* **25**, 1253–1264 (2019).

47. Dioguardi, M. *et al.* The Association between Tooth Loss and Alzheimer’s Disease: a Systematic Review with Meta-Analysis of Case Control Studies. *Dent. J.* **7**, 49 (2019).

48. Cao, R. *et al.* Effect of non-surgical periodontal therapy on glycemic control of type 2 diabetes mellitus: a systematic review and Bayesian network meta-analysis. *BMC Oral Health* **19**, 176 (2019).

49. Corbella, S., Francetti, L., Taschieri, S., De Siena, F. & Fabbro, M. D. Effect of periodontal treatment on glycemic control of patients with diabetes: A systematic review and meta-analysis. *J. Diabetes Investig.* **4**, 502–509 (2013).

50. Wang, Q., Kang, J., Cai, X., Wu, Y. & Zhao, L. The association between chronic periodontitis and vasculogenic erectile dysfunction: a systematic review and meta-analysis. *J. Clin. Periodontol.* **43**, 206–215 (2016).

51. Gopinath, D., Kunnath Menon, R., K. Veettil, S., George Botelho, M. & Johnson, N. W. Periodontal Diseases as Putative Risk Factors for Head and Neck Cancer: Systematic Review and Meta-Analysis. *Cancers* **12**, 1893 (2020).

52. Rapone, B. *et al.* Does Periodontal Inflammation Affect Type 1 Diabetes in Childhood and Adolescence? A Meta-Analysis. *Front. Endocrinol.* **11**, 278 (2020).

53. Wu, C. *et al.* Epidemiologic relationship between periodontitis and type 2 diabetes mellitus. *BMC Oral Health* **20**, 204 (2020).

54. Botero, J. E., Rodríguez‐Medina, C., Jaramillo‐Echeverry, A. & Contreras, A. Association between human cytomegalovirus and periodontitis: A systematic review and meta‐analysis. *J. Periodontal Res.* **55**, 551–558 (2020).

55. Zhang, X., Gu, H., Xie, S. & Su, Y. Periodontitis in patients with psoriasis: A systematic review and meta‐analysis. *Oral Dis.* **28**, 33–43 (2022).

56. Schmitt, A., Carra, M. C., Boutouyrie, P. & Bouchard, P. Periodontitis and arterial stiffness: a systematic review and meta-analysis. *J. Clin. Periodontol.* **42**, 977–987 (2015).

57. Atieh, M. A., M. Faggion, C. & Seymour, G. J. Cytokines in patients with type 2 diabetes and chronic periodontitis: A systematic review and meta-analysis. *Diabetes Res. Clin. Pract.* **104**, e38–e45 (2014).

58. Adverse pregnancy outcomes and periodontitis: A systematic review and meta-analysis exploring potential association. *Quintessence Int.* **47**, 193–204 (2016).

59. Hua, F. *et al.* Oral hygiene care for critically ill patients to prevent ventilator-associated pneumonia. *Cochrane Database Syst. Rev.* (2016) doi:10.1002/14651858.CD008367.pub3.

60. Araújo, M. M. *et al.* Association between depression and periodontitis: a systematic review and meta-analysis. *J. Clin. Periodontol.* **43**, 216–228 (2016).

61. Ziukaite, L., Slot, D. E. & Van der Weijden, F. A. Prevalence of diabetes mellitus in people clinically diagnosed with periodontitis: A systematic review and meta-analysis of epidemiologic studies. *J. Clin. Periodontol.* **45**, 650–662 (2018).

62. Ferreira, M. K. M. *et al.* Is there an association between asthma and periodontal disease among adults? Systematic review and meta-analysis. *Life Sci.* **223**, 74–87 (2019).

63. Teeuw, W. J. *et al.* Treatment of periodontitis improves the atherosclerotic profile: a systematic review and meta-analysis. *J. Clin. Periodontol.* **41**, 70–79 (2014).

64. Papageorgiou, S. N., Reichert, C., Jäger, A. & Deschner, J. Effect of overweight/obesity on response to periodontal treatment: systematic review and a meta-analysis. *J. Clin. Periodontol.* **42**, 247–261 (2015).

65. Tomás, I., Diz, P., Tobías, A., Scully, C. & Donos, N. Periodontal health status and bacteraemia from daily oral activities: systematic review/meta-analysis. *J. Clin. Periodontol.* **39**, 213–228 (2012).

66. Peng, J. *et al.* The relationship between tooth loss and mortality from all causes, cardiovascular diseases, and coronary heart disease in the general population: systematic review and dose–response meta-analysis of prospective cohort studies. *Biosci. Rep.* **39**, BSR20181773 (2019).

67. Ferreira, R. de O. *et al.* Physical Activity Reduces the Prevalence of Periodontal Disease: Systematic Review and Meta-Analysis. *Front. Physiol.* **10**, 234 (2019).

68. da Silva, T. A., Abreu, L. G. & Esteves Lima, R. P. A meta‐analysis on the effect of periodontal treatment on the glomerular filtration rate of chronic kidney disease individuals: A systematic review and meta‐analysis was conducted to assess the impact of the periodontal treatment on the glomerular filtration rate of individuals with chronic kidney disease. *Spec. Care Dentist.* **41**, 670–678 (2021).

69. Daudt, L. D. *et al.* Association between metabolic syndrome and periodontitis: a systematic review and meta-analysis. *Braz. Oral Res.* **32**, (2018).

70. Zhao, D. *et al.* The directional and non-directional associations of periodontitis with chronic kidney disease: A systematic review and meta-analysis of observational studies. *J. Periodontal Res.* **53**, 682–704 (2018).

71. Moraschini, V., Barboza, E. S. P. & Peixoto, G. A. The impact of diabetes on dental implant failure: a systematic review and meta-analysis. *Int. J. Oral Maxillofac. Surg.* **45**, 1237–1245 (2016).

72. Leira, Y. *et al.* Is Periodontal Disease Associated with Alzheimer’s Disease? A Systematic Review with Meta-Analysis. *Neuroepidemiology* **48**, 21–31 (2017).

73. Moraschini, V., de Albuquerque Calasans-Maia, J. & Diuana Calasans-Maia, M. Association Between Asthma and Periodontal Disease: A Systematic Review and Meta-Analysis. *J. Periodontol.* 1–20 (2017) doi:10.1902/jop.2017.170363.

74. Joshi, C. *et al.* Detection of periodontal microorganisms in coronary atheromatous plaque specimens of myocardial infarction patients: A systematic review and meta-analysis. *Trends Cardiovasc. Med.* **31**, 69–82 (2021).

75. Conde-Agudelo, A., Villar, J. & Lindheimer, M. Maternal infection and risk of preeclampsia: Systematic review and metaanalysis. *Am. J. Obstet. Gynecol.* **198**, 7–22 (2008).

76. Simpson, T. C., Needleman, I., Wild, S. H., Moles, D. R. & Mills, E. J. Treatment of periodontal disease for glycaemic control in people with diabetes. in *Cochrane Database of Systematic Reviews* (ed. The Cochrane Collaboration) CD004714.pub2 (John Wiley & Sons, Ltd, 2010). doi:10.1002/14651858.CD004714.pub2.

77. Boutin, A. *et al.* Treatment of Periodontal Disease and Prevention of Preterm Birth: Systematic Review and Meta-analysis. *Am. J. Perinatol.* **30**, 537–544 (2012).

78. Suvan, J., D’Aiuto, F., Moles, D. R., Petrie, A. & Donos, N. Association between overweight/obesity and periodontitis in adults. A systematic review: Hypothalamic obesity. *Obes. Rev.* **12**, e381–e404 (2011).

79. Moura-Grec, P. G. de, Marsicano, J. A., Carvalho, C. A. P. de & Sales-Peres, S. H. de C. Obesity and periodontitis: systematic review and meta-analysis. *Ciênc. Saúde Coletiva* **19**, 1763–1772 (2014).

80. Machado, V. *et al.* Periodontitis Impact in Interleukin-6 Serum Levels in Solid Organ Transplanted Patients: A Systematic Review and Meta-Analysis. *Diagnostics* **10**, 184 (2020).

81. de Oliveira Ferreira, R. *et al.* Does periodontitis represent a risk factor for rheumatoid arthritis? A systematic review and meta-analysis. *Ther. Adv. Musculoskelet. Dis.* **11**, 1759720X1985851 (2019).

82. Monje, A., Catena, A. & Borgnakke, W. S. Association between diabetes mellitus/hyperglycaemia and peri-implant diseases: Systematic review and meta-analysis. *J. Clin. Periodontol.* **44**, 636–648 (2017).

83. Martens, L., De Smet, S., Yusof, M. Y. P. M. & Rajasekharan, S. Association between overweight/obesity and periodontal disease in children and adolescents: a systematic review and meta-analysis. *Eur. Arch. Paediatr. Dent.* **18**, 69–82 (2017).

84. Esteves Lima, R. P. *et al.* Association Between Periodontitis and Gestational Diabetes Mellitus: Systematic Review and Meta-Analysis. *J. Periodontol.* **87**, 48–57 (2016).

85. Li, W. *et al.* Is periodontal disease a risk indicator for colorectal cancer? A systematic review and meta‐analysis. *J. Clin. Periodontol.* **48**, 336–347 (2021).

86. Rosa, M. I. da, Pires, P. D. S., Medeiros, L. R., Edelweiss, M. I. & Martínez-Mesa, J. Periodontal disease treatment and risk of preterm birth: a systematic review and meta-analysis. *Cad. Saúde Pública* **28**, 1823–1833 (2012).

87. Leira, Y. *et al.* Association between periodontitis and ischemic stroke: a systematic review and meta-analysis. *Eur. J. Epidemiol.* **32**, 43–53 (2017).

88. Hussain, S. B. *et al.* Is there a bidirectional association between rheumatoid arthritis and periodontitis? A systematic review and meta-analysis. *Semin. Arthritis Rheum.* **50**, 414–422 (2020).

89. Botelho, J. *et al.* Stress, salivary cortisol and periodontitis: A systematic review and meta-analysis of observational studies. *Arch. Oral Biol.* **96**, 58–65 (2018).

90. Zheng, D. *et al.* Periodontal disease and emotional disorders: A meta‐analysis. *J. Clin. Periodontol.* **48**, 180–204 (2021).

91. Chambrone, L., Pannuti, C. M., Guglielmetti, M. R. & Chambrone, L. A. Evidence grade associating periodontitis with preterm birth and/or low birth weight: II. A systematic review of randomized trials evaluating the effects of periodontal treatment: Periodontitis and adverse pregnancy outcomes. *J. Clin. Periodontol.* **38**, 902–914 (2011).

92. Jain, A. *et al.* Effect of scaling and root planing as monotherapy on glycemic control in patients of Type 2 diabetes with chronic periodontitis: A systematic review and meta-analysis. *J. Indian Soc. Periodontol.* **23**, 303 (2019).

93. Martin-Cabezas, R. *et al.* Association between periodontitis and arterial hypertension: A systematic review and meta-analysis. *Am. Heart J.* **180**, 98–112 (2016).

94. Shang, R. & Gao, L. Impact of hyperglycemia on the rate of implant failure and peri-implant parameters in patients with type 2 diabetes mellitus. *J. Am. Dent. Assoc.* **152**, 189-201.e1 (2021).

95. Chambrone, L. *et al.* Periodontitis and chronic kidney disease: a systematic review of the association of diseases and the effect of periodontal treatment on estimated glomerular filtration rate. *J. Clin. Periodontol.* **40**, 443–456 (2013).

96. Chambrone, L., Guglielmetti, M. R., Pannuti, C. M. & Chambrone, L. A. Evidence grade associating periodontitis to preterm birth and/or low birth weight: I. A systematic review of prospective cohort studies: Periodontitis and adverse pregnancy outcomes. *J. Clin. Periodontol.* **38**, 795–808 (2011).

97. Paraskevas, S., Huizinga, J. D. & Loos, B. G. A systematic review and meta-analyses on C-reactive protein in relation to periodontitis. *J. Clin. Periodontol.* **35**, 277–290 (2008).

98. Fagundes, N. C. F. *et al.* Periodontitis As A Risk Factor For Stroke: A Systematic Review And Meta-Analysis. *Vasc. Health Risk Manag.* **Volume 15**, 519–532 (2019).

99. da Silva, H. E. C. *et al.* Effect of intra-pregnancy nonsurgical periodontal therapy on inflammatory biomarkers and adverse pregnancy outcomes: a systematic review with meta-analysis. *Syst. Rev.* **6**, 197 (2017).

100. Bi, W. G., Emami, E., Luo, Z.-C., Santamaria, C. & Wei, S. Q. Effect of periodontal treatment in pregnancy on perinatal outcomes: a systematic review and meta-analysis. *J. Matern.-Fetal Neonatal Med. Off. J. Eur. Assoc. Perinat. Med. Fed. Asia Ocean. Perinat. Soc. Int. Soc. Perinat. Obstet.* 1–10 (2019) doi:10.1080/14767058.2019.1678142.

101. Cabanillas‐Balsera, D. *et al.* Association between diabetes and nonretention of root filled teeth: a systematic review and meta‐analysis. *Int. Endod. J.* **52**, 297–306 (2019).

102. Nibali, L. *et al.* Association Between Metabolic Syndrome and Periodontitis: A Systematic Review and Meta-analysis. *J. Clin. Endocrinol. Metab.* **98**, 913–920 (2013).

103. Gomes‐Filho, I. S. *et al.* Periodontitis and respiratory diseases: A systematic review with meta‐analysis. *Oral Dis.* **26**, 439–446 (2020).

104. Kim, A. J., Lo, A. J., Pullin, D. A., Thornton-Johnson, D. S. & Karimbux, N. Y. Scaling and Root Planing Treatment for Periodontitis to Reduce Preterm Birth and Low Birth Weight: A Systematic Review and Meta-Analysis of Randomized Controlled Trials. *J. Periodontol.* **83**, 1508–1519 (2012).

105. Maldonado, A., Laugisch, O., Bürgin, W., Sculean, A. & Eick, S. Clinical periodontal variables in patients with and without dementia—a systematic review and meta-analysis. *Clin. Oral Investig.* **22**, 2463–2474 (2018).

106. Corbella, S. *et al.* Is periodontitis a risk indicator for cancer? A meta-analysis. *PLOS ONE* **13**, e0195683 (2018).

107. Chrcanovic, B. R., Albrektsson, T. & Wennerberg, A. Diabetes and Oral Implant Failure: A Systematic Review. *J. Dent. Res.* **93**, 859–867 (2014).

108. Tang, Q. *et al.* A Possible Link Between Rheumatoid Arthritis and Periodontitis: A Systematic Review and Meta-analysis. *Int. J. Periodontics Restorative Dent.* **37**, 79–86 (2017).

109. Deng, L., Li, C., Li, Q., Zhang, Y. & Zhao, H. [Periodontal treatment for cardiovascular risk factors: a systematic review]. *Hua Xi Kou Qiang Yi Xue Za Zhi Huaxi Kouqiang Yixue Zazhi West China J. Stomatol.* **31**, 463–467 (2013).

110. Gupta, A., Aggarwal, V., Mehta, N., Abraham, D. & Singh, A. Diabetes mellitus and the healing of periapical lesions in root filled teeth: a systematic review and meta‐analysis. *Int. Endod. J.* **53**, 1472–1484 (2020).

111. Kaur, S., Bright, R., Proudman, S. M. & Bartold, P. M. Does periodontal treatment influence clinical and biochemical measures for rheumatoid arthritis? A systematic review and meta-analysis. *Semin. Arthritis Rheum.* **44**, 113–122 (2014).

112. Gomes, M. S. *et al.* Can Apical Periodontitis Modify Systemic Levels of Inflammatory Markers? A Systematic Review and Meta-analysis. *J. Endod.* **39**, 1205–1217 (2013).

113. Wang, W. *et al.* Association between Periodontitis and Carotid Artery Calcification: A Systematic Review and Meta-Analysis. *BioMed Res. Int.* **2021**, 1–9 (2021).

114. Esteves Lima, R. P., Atanazio, A. R. S., Costa, F. O., Cunha, F. A. & Abreu, L. G. IMPACT OF NON-SURGICAL PERIODONTAL TREATMENT ON SERUM TNF-α LEVELS IN INDIVIDUALS WITH TYPE 2 DIABETES: A SYSTEMATIC REVIEW AND META-ANALYSIS. *J. Evid. Based Dent. Pract.* **21**, 101546 (2021).

115. Abariga, S. A. & Whitcomb, B. W. Periodontitis and gestational diabetes mellitus: a systematic review and meta-analysis of observational studies. *BMC Pregnancy Childbirth* **16**, 344 (2016).

116. Engebretson, S. & Kocher, T. Evidence that periodontal treatment improves diabetes outcomes: a systematic review and meta-analysis. *J. Periodontol.* **84**, S153–S163 (2013).

117. Figuero, E., Carrillo-de-Albornoz, A., Martín, C., Tobías, A. & Herrera, D. Effect of pregnancy on gingival inflammation in systemically healthy women: a systematic review. *J. Clin. Periodontol.* **40**, 457–473 (2013).

118. Kunnen, A. *et al.* Review Article: Periodontal disease and pre-eclampsia: a systematic review: Periodontal disease and pre-eclampsia. *J. Clin. Periodontol.* **37**, 1075–1087 (2010).

119. Machado, V., Escalda, C., Proença, L., Mendes, J. J. & Botelho, J. Is There a Bidirectional Association between Polycystic Ovarian Syndrome and Periodontitis? A Systematic Review and Meta-analysis. *J. Clin. Med.* **9**, E1961 (2020).

120. Georgiou, A. C., Crielaard, W., Armenis, I., de Vries, R. & van der Waal, S. V. Apical Periodontitis Is Associated with Elevated Concentrations of Inflammatory Mediators in Peripheral Blood: A Systematic Review and Meta-analysis. *J. Endod.* **45**, 1279-1295.e3 (2019).

121. Garde, S., Akhter, R., Nguyen, M. A., Chow, C. K. & Eberhard, J. Periodontal Therapy for Improving Lipid Profiles in Patients with Type 2 Diabetes Mellitus: A Systematic Review and Meta-Analysis. *Int. J. Mol. Sci.* **20**, 3826 (2019).

122. Lorenzo-Pouso, A. I., Castelo-Baz, P., Rodriguez-Zorrilla, S., Pérez-Sayáns, M. & Vega, P. Association between periodontal disease and inflammatory bowel disease: a systematic review and meta-analysis. *Acta Odontol. Scand.* **79**, 344–353 (2021).

123. Romandini, M. *et al.* Periodontitis, Edentulism, and Risk of Mortality: A Systematic Review with Meta-analyses. *J. Dent. Res.* **100**, 37–49 (2021).

124. Artese, H. P. C. *et al.* Periodontal Therapy and Systemic Inflammation in Type 2 Diabetes Mellitus: A Meta-Analysis. *PLOS ONE* **10**, e0128344 (2015).

125. Segura-Egea, J. J. *et al.* Association between diabetes and the prevalence of radiolucent periapical lesions in root-filled teeth: systematic review and meta-analysis. *Clin. Oral Investig.* **20**, 1133–1141 (2016).

126. Chen, Y. *et al.* Baseline HbA1c Level Influences the Effect of Periodontal Therapy on Glycemic Control in People with Type 2 Diabetes and Periodontitis: A Systematic Review on Randomized Controlled Trails. *Diabetes Ther.* **12**, 1249–1278 (2021).

127. Maisonneuve, P., Amar, S. & Lowenfels, A. B. Periodontal disease, edentulism, and pancreatic cancer: a meta-analysis. *Ann. Oncol. Off. J. Eur. Soc. Med. Oncol.* **28**, 985–995 (2017).

128. Bouziane, A., Ahid, S., Abouqal, R. & Ennibi, O. Effect of periodontal therapy on prevention of gastric *Helicobacter pylori* recurrence: a systematic review and meta-analysis. *J. Clin. Periodontol.* **39**, 1166–1173 (2012).

129. Darré, L., Vergnes, J.-N., Gourdy, P. & Sixou, M. Efficacy of periodontal treatment on glycaemic control in diabetic patients: A meta-analysis of interventional studies. *Diabetes Metab.* **34**, 497–506 (2008).

130. Machado, V. *et al.* Serum C-Reactive Protein and Periodontitis: A Systematic Review and Meta-Analysis. *Front. Immunol.* **12**, 706432 (2021).

131. Wu, Y. *et al.* Hematopoietic and lymphatic cancers in patients with periodontitis: a systematic review and meta-analysis. *Med. Oral Patol. Oral Cirugia Bucal* e21–e28 (2020) doi:10.4317/medoral.23166.

132. Lü, Z. *et al.* [Periodontal therapy for rheumatoid arthritis: a systematic review]. *Hua Xi Kou Qiang Yi Xue Za Zhi Huaxi Kouqiang Yixue Zazhi West China J. Stomatol.* **29**, 375–378 (2011).

133. Corbella, S., Taschieri, S., Francetti, L., De Siena, F. & Del Fabbro, M. Periodontal disease as a risk factor for adverse pregnancy outcomes: a systematic review and meta-analysis of case–control studies. *Odontology* **100**, 232–240 (2012).

134. Wijarnpreecha, K. *et al.* The Association between Periodontitis and Nonalcoholic Fatty Liver Disease: A Systematic Review and Meta-analysis. *J. Gastrointestin. Liver Dis.* **29**, 211–217 (2020).

135. Lorenzo‐Pouso, A. I. *et al.* Association between periodontitis and medication‐related osteonecrosis of the jaw: A systematic review and meta‐analysis. *J. Oral Pathol. Med.* **49**, 190–200 (2020).

136. Demmer, R. T. *et al.* The Influence of Anti-Infective Periodontal Treatment on C-Reactive Protein: A Systematic Review and Meta-Analysis of Randomized Controlled Trials. *PLoS ONE* **8**, e77441 (2013).

137. Polyzos, N. P. *et al.* Obstetric outcomes after treatment of periodontal disease during pregnancy: systematic review and meta-analysis. *BMJ* **341**, c7017–c7017 (2010).

138. Martorano-Fernandes, L. *et al.* Oral candidiasis and denture stomatitis in diabetic patients: Systematic review and meta-analysis. *Braz. Oral Res.* **34**, e113 (2020).

139. Peña, D. E. R., Innocentini, L. M. A. R., Saraiva, M. C. P., Lourenço, A. G. & Motta, A. C. F. Oral candidiasis prevalence in human immunodeficiency virus-1 and pulmonary tuberculosis coinfection: A systematic review and meta-analysis. *Microb. Pathog.* **150**, 104720 (2021).

140. Botelho, J. *et al.* Periodontitis and circulating blood cell profiles: a systematic review and meta-analysis. *Exp. Hematol.* **93**, 1–13 (2021).

141. Roca-Millan, E. *et al.* Periodontal treatment on patients with cardiovascular disease: Systematic review and meta-analysis. *Med. Oral Patol. Oral Cirugia Bucal* 0–0 (2018) doi:10.4317/medoral.22725.

142. Jensen, E., Allen, G., Bednarz, J., Couper, J. & Peña, A. Periodontal risk markers in children and adolescents with type 1 diabetes: A systematic review and meta‐analysis. *Diabetes Metab. Res. Rev.* **37**, (2021).

143. Manrique‐Corredor, E. J. *et al.* Maternal periodontitis and preterm birth: Systematic review and meta‐analysis. *Community Dent. Oral Epidemiol.* **47**, 243–251 (2019).

144. Ungprasert, P., Wijarnpreecha, K. & Wetter, D. A. Periodontitis and risk of psoriasis: a systematic review and meta-analysis. *J. Eur. Acad. Dermatol. Venereol.* **31**, 857–862 (2017).

145. Liu, L. H. *et al.* Chronic periodontitis and the risk of erectile dysfunction: a systematic review and meta-analysis. *Int. J. Impot. Res.* **29**, 43–48 (2017).

146. Teeuw, W. J., Gerdes, V. E. A. & Loos, B. G. Effect of Periodontal Treatment on Glycemic Control of Diabetic Patients. *Diabetes Care* **33**, 421–427 (2010).

147. Farook, F., Al Meshrafi, A., Mohamed Nizam, N. & Al Shammari, A. The Association Between Periodontitis and Erectile Dysfunction: A Systematic Review and Meta-Analysis. *Am. J. Mens Health* **15**, 155798832110072 (2021).

148. Wang, X., Han, X., Guo, X., Luo, X. & Wang, D. The Effect of Periodontal Treatment on Hemoglobin A1c Levels of Diabetic Patients: A Systematic Review and Meta-Analysis. *PLoS ONE* **9**, e108412 (2014).

149. Hu, X., Zhang, J., Qiu, Y. & Liu, Z. Periodontal disease and the risk of Alzheimer’s disease and mild cognitive impairment: a systematic review and meta‐analysis. *Psychogeriatrics* **21**, 813–825 (2021).

150. Schwartz, M., Acosta, L., Hung, Y.-L., Padilla, M. & Enciso, R. Effects of CPAP and mandibular advancement device treatment in obstructive sleep apnea patients: a systematic review and meta-analysis. *Sleep Breath.* **22**, 555–568 (2018).

151. Bahekar, A. A., Singh, S., Saha, S., Molnar, J. & Arora, R. The prevalence and incidence of coronary heart disease is significantly increased in periodontitis: A meta-analysis. *Am. Heart J.* **154**, 830–837 (2007).

152. Hsu, Y., Nair, M., Angelov, N., Lalla, E. & Lee, C. Impact of diabetes on clinical periodontal outcomes following non‐surgical periodontal therapy. *J. Clin. Periodontol.* **46**, 206–217 (2019).

153. Fuggle, N. R., Smith, T. O., Kaul, A. & Sofat, N. Hand to Mouth: A Systematic Review and Meta-Analysis of the Association between Rheumatoid Arthritis and Periodontitis. *Front. Immunol.* **7**, (2016).

154. Stöhr, J., Barbaresko, J., Neuenschwander, M. & Schlesinger, S. Bidirectional association between periodontal disease and diabetes mellitus: a systematic review and meta-analysis of cohort studies. *Sci. Rep.* **11**, 13686 (2021).

155. Ratz, T. *et al.* A possible link between ankylosing spondylitis and periodontitis: a systematic review and meta-analysis. *Rheumatology* **54**, 500–510 (2015).

156. Wu, D. *et al.* Decreased Hemoglobin Concentration and Iron Metabolism Disorder in Periodontitis: Systematic Review and Meta-Analysis. *Front. Physiol.* **10**, 1620 (2020).

157. Al-Jewair, T. S., Al-Jasser, R. & Almas, K. Periodontitis and obstructive sleep apnea’s bidirectional relationship: a systematic review and meta-analysis. *Sleep Breath.* **19**, 1111–1120 (2015).

158. Chaffee, B. W. & Weston, S. J. Association Between Chronic Periodontal Disease and Obesity: A Systematic Review and Meta-Analysis. *J. Periodontol.* **81**, 1708–1724 (2010).

159. da Silva, F. G., Pola, N. M., Casarin, M., Silva, C. F. e & Muniz, F. W. M. G. Association between clinical measures of gingival inflammation and obesity in adults: systematic review and meta-analyses. *Clin. Oral Investig.* **25**, 4281–4298 (2021).

160. Larvin, H., Kang, J., Aggarwal, V. R., Pavitt, S. & Wu, J. Risk of incident cardiovascular disease in people with periodontal disease: A systematic review and meta‐analysis. *Clin. Exp. Dent. Res.* **7**, 109–122 (2021).

161. Nepomuceno, R. *et al.* Serum lipid levels in patients with periodontal disease: A meta-analysis and meta-regression. *J. Clin. Periodontol.* **44**, 1192–1207 (2017).

162. Qiao, Y. *et al.* Rheumatoid arthritis risk in periodontitis patients: A systematic review and meta-analysis. *Joint Bone Spine* **87**, 556–564 (2020).

163. Dicembrini, I. *et al.* Type 1 diabetes and periodontitis: prevalence and periodontal destruction—a systematic review. *Acta Diabetol.* **57**, 1405–1412 (2020).

164. Xiao, L., Zhang, Q., Peng, Y., Wang, D. & Liu, Y. The effect of periodontal bacteria infection on incidence and prognosis of cancer: A systematic review and meta-analysis. *Medicine (Baltimore)* **99**, e19698 (2020).

165. Yang, S. *et al.* Association between periodontitis and peripheral artery disease: a systematic review and meta-analysis. *BMC Cardiovasc. Disord.* **18**, 141 (2018).

166. Orlandi, M. *et al.* Association between periodontal disease and its treatment, flow-mediated dilatation and carotid intima-media thickness: A systematic review and meta-analysis. *Atherosclerosis* **236**, 39–46 (2014).

167. Zhang, Y. *et al.* The Association between Periodontitis and Inflammatory Bowel Disease: A Systematic Review and Meta-analysis. *BioMed Res. Int.* **2021**, 1–8 (2021).

168. Zhang, J. *et al.* Influence of anti‐rheumatic agents on the periodontal condition of patients with rheumatoid arthritis and periodontitis: A systematic review and meta‐analysis. *J. Periodontal Res.* **56**, 1099–1115 (2021).

169. Moliner-Sánchez, C. A. *et al.* Effect of per Capita Income on the Relationship between Periodontal Disease during Pregnancy and the Risk of Preterm Birth and Low Birth Weight Newborn. Systematic Review and Meta-Analysis. *Int. J. Environ. Res. Public. Health* **17**, 8015 (2020).

170. Baeza, M. *et al.* Effect of periodontal treatment in patients with periodontitis and diabetes: systematic review and meta-analysis. *J. Appl. Oral Sci.* **28**, e20190248 (2020).

171. Rutter-Locher, Z., Smith, T. O., Giles, I. & Sofat, N. Association between Systemic Lupus Erythematosus and Periodontitis: A Systematic Review and Meta-analysis. *Front. Immunol.* **8**, 1295 (2017).

172. Chen, Y., Zhu, B., Wu, C., Lin, R. & Zhang, X. Periodontal Disease and Tooth Loss Are Associated with Lung Cancer Risk. *BioMed Res. Int.* **2020**, 1–12 (2020).

173. Koletsi, D., Iliadi, A., Tzanetakis, G. N., Vavuranakis, M. & Eliades, T. Cardiovascular Disease and Chronic Endodontic Infection. Is There an Association? A Systematic Review and Meta-Analysis. *Int. J. Environ. Res. Public. Health* **18**, 9111 (2021).

174. Ozturk, A. Periodontal Treatment Is Associated With Improvement in Gastric Helicobacter pylori Eradication: An Updated Meta-analysis of Clinical Trials. *Int. Dent. J.* **71**, 188–196 (2021).

175. Zhou, X., Cao, F., Lin, Z. & Wu, D. Updated Evidence of Association Between Periodontal Disease and Incident Erectile Dysfunction. *J. Sex. Med.* **16**, 61–69 (2019).

176. Lv, X. *et al.* Periodontal Disease and Age-Related Macular Degeneration: A Meta-Analysis of 112,240 Participants. *BioMed Res. Int.* **2020**, 1–11 (2020).

177. Xu, J. & Duan, X. Association between periodontitis and hyperlipidaemia: A systematic review and meta‐analysis. *Clin. Exp. Pharmacol. Physiol.* **47**, 1861–1873 (2020).

178. Silva, D. S. *et al.* Evidence‐Based Research on Effectiveness of Periodontal Treatment in Rheumatoid Arthritis Patients: A Systematic Review and Meta‐Analysis. *Arthritis Care Res.* **74**, 1723–1735 (2022).

179. Calderaro, D. C. *et al.* Influência do tratamento periodontal na artrite reumatoide: revisão sistemática e metanálise. *Rev. Bras. Reumatol.* **57**, 238–244 (2017).

180. Ren, Q., Yan, X., Zhou, Y. & Li, W. X. Periodontal therapy as adjunctive treatment for gastric *Helicobacter pylori* infection. *Cochrane Database Syst. Rev.* **2016**, (2016).

181. Zainal Abidin, Z. *et al.* Periodontal health status of children and adolescents with diabetes mellitus: a systematic review and meta‐analysis. *Aust. Dent. J.* **66**, (2021).

182. Hussain, S. B. *et al.* Periodontitis and Systemic Lupus Erythematosus: A systematic review and meta‐analysis. *J. Periodontal Res.* **57**, 1–10 (2022).

183. Mirzaei, A. *et al.* Association of hyperglycaemia and periodontitis: an updated systematic review and meta-analysis. *J. Diabetes Metab. Disord.* **20**, 1327–1336 (2021).

184. Maarse, F. *et al.* Sjögren’s syndrome is not a risk factor for periodontal disease: a systematic review. *Clin. Exp. Rheumatol.* **37 Suppl 118**, 225–233 (2019).

185. Souza, M. L., Massignan, C., Glazer Peres, K. & Aurélio Peres, M. Association between metabolic syndrome and tooth loss. *J. Am. Dent. Assoc.* **150**, 1027-1039.e7 (2019).

186. Jalili, M., Mahmoodabadi, K. A. & Sayehmiri, K. Relationship between Helicobacter pylori and Periodontal Diseases: A Meta-Analysis Study and Systematic Review. *Open Dent. J.* **14**, 362–368 (2020).

187. Silveira, A. L. N. de M. e S., Magno, M. B. & Soares, T. R. C. The relationship between special needs and dental trauma. A systematic review and meta‐analysis. *Dent. Traumatol.* **36**, 218–236 (2020).

188. Aminoshariae, A., Kulild, J. & Fouad, A. F. The impact of cardiovascular disease and endodontic outcome: a systematic review of longitudinal studies. *Clin. Oral Investig.* **24**, 3813–3819 (2020).

189. Aminoshariae, A., Kulild, J. C. & Fouad, A. F. The Impact of Endodontic Infections on the Pathogenesis of Cardiovascular Disease(s): A Systematic Review with Meta-analysis Using GRADE. *J. Endod.* **44**, 1361-1366.e3 (2018).

190. Alvarenga, M. O. P. *et al.* Masticatory Dysfunction by Extensive Tooth Loss as a Risk Factor for Cognitive Deficit: A Systematic Review and Meta-Analysis. *Front. Physiol.* **10**, 832 (2019).

191. Araújo, B. C. L., de Magalhães Simões, S., de Gois-Santos, V. T. & Martins-Filho, P. R. S. Association Between Mouth Breathing and Asthma: a Systematic Review and Meta-analysis. *Curr. Allergy Asthma Rep.* **20**, 24 (2020).

192. Hermont, A. P. *et al.* Tooth Erosion and Eating Disorders: A Systematic Review and Meta-Analysis. *PLoS ONE* **9**, e111123 (2014).

193. Lockhart, P. B. *et al.* Effect of dental treatment before cardiac valve surgery. *J. Am. Dent. Assoc.* **150**, 739-747.e9 (2019).

194. Wang, T. *et al.* Periodontal disease and cognitive deficits: A systematic review and meta-analysis. *Neurol. Asia* 12 (2020).

195. Jiang, X., Zhu, Y., Liu, Z., Tian, Z. & Zhu, S. Association between diabetes and dental implant complications: a systematic review and meta-analysis. *Acta Odontol. Scand.* **79**, 9–18 (2021).

196. Fang, W. *et al.* Tooth loss as a risk factor for dementia: systematic review and meta-analysis of 21 observational studies. *BMC Psychiatry* **18**, 345 (2018).

197. Souto-Souza, D. *et al.* Is there an association between attention deficit hyperactivity disorder in children and adolescents and the occurrence of bruxism? A systematic review and meta-analysis. *Sleep Med. Rev.* **53**, 101330 (2020).

198. Ríos-Osorio, N. *et al.* Association between type 2 diabetes mellitus and the evolution of endodontic pathology. *Quintessence Int. Berl. Ger. 1985* **51**, 100–107 (2020).

199. Blaizot, A., Vergnes, J.-N., Nuwwareh, S., Amar, J. & Sixou, M. Periodontal diseases and cardiovascular events: meta-analysis of observational studies. *Int. Dent. J.* **59**, 197–209 (2009).

200. Oh, B. *et al.* Association between residual teeth number in later life and incidence of dementia: A systematic review and meta-analysis. *BMC Geriatr.* **18**, 48 (2018).

201. AlOtaibi, A., Ben Shaber, S., AlBatli, A., AlGhamdi, T. & Murshid, E. A systematic review of population-based gingival health studies among children and adolescents with autism spectrum disorder. *Saudi Dent. J.* **33**, 370–374 (2021).

202. Easwaran, H. N. *et al.* Early Childhood Caries and Iron Deficiency Anaemia: A Systematic Review and Meta-Analysis. *Caries Res.* **56**, 36–46 (2022).

203. Ji, S.-Q. *et al.* Iron deficiency and early childhood caries: a systematic review and meta-analysis. *Chin. Med. J. (Engl.)* **134**, 2832–2837 (2021).

204. Sun, X.-N., Zhou, J.-B. & Li, N. Poor Oral Health in Patients with Schizophrenia: a Meta-Analysis of Case-Control Studies. *Psychiatr. Q.* **92**, 135–145 (2021).

205. Drumond, V. Z. *et al.* Dental Caries in Children with Attention Deficit/Hyperactivity Disorder: A Meta-Analysis. *Caries Res.* **56**, 3–14 (2022).

206. Le, Q.-A. *et al.* Does Treatment of Gingivitis During Pregnancy Improve Pregnancy Outcomes? A Systematic Review and Meta-Analysis. *Oral Health Prev. Dent.* **19**, 565–572 (2021).

207. Marzouk, T. *et al.* Association between oral clefts and periodontal clinical measures: A meta‐analysis. *Int. J. Paediatr. Dent.* **32**, 558–575 (2022).

208. Del Rei Daltro Rosa, C. D. *et al.* Does non-surgical periodontal treatment influence on rheumatoid arthritis? A systematic review and meta-analysis. *Saudi Dent. J.* **33**, 795–804 (2021).

209. Porto, E. C. L. *et al.* Periodontite materna e baixo peso ao nascer: revisão sistemática e metanálise. *Ciênc. Saúde Coletiva* **26**, 5383–5392 (2021).

210. Orlandi, M. *et al.* Impact of the treatment of periodontitis on systemic health and quality of life: A systematic review. *J. Clin. Periodontol.* jcpe.13554 (2021) doi:10.1111/jcpe.13554.

211. Serni, L. *et al.* Association between chronic kidney disease and periodontitis. A systematic review and metanalysis. *Oral Dis.* odi.14062 (2021) doi:10.1111/odi.14062.

212. Andrade, C. A. S. *et al.* Survival rate and peri-implant evaluation of immediately loaded dental implants in individuals with type 2 diabetes mellitus: a systematic review and meta-analysis. *Clin. Oral Investig.* **26**, 1797–1810 (2022).

213. Gusman, D. J. R. *et al.* Periodontal disease severity in subjects with dementia: A systematic review and meta-analysis. *Arch. Gerontol. Geriatr.* **76**, 147–159 (2018).

214. Pi, X. *et al.* A Meta-Analysis of Oral Health Status of Children with Autism. *J. Clin. Pediatr. Dent.* **44**, 1–7 (2020).

215. Zhang, Y., Lin, L., Liu, J., Shi, L. & Lu, J. Dental Caries Status in Autistic Children: A Meta-analysis. *J. Autism Dev. Disord.* **50**, 1249–1257 (2020).

216. Hatipoğlu, Ö., Önsüren, A. S., Hatipoğlu, F. P. & Kurt, A. Caries‐related salivary parameters and oral microbial flora in patients with type 1 diabetes: A meta‐analysis. *Diabetes Metab. Res. Rev.* **38**, (2022).

217. Xi, W. *et al.* [Oral health status of patients undergoing hemodialysis: a Meta-analysis]. *Hua Xi Kou Qiang Yi Xue Za Zhi Huaxi Kouqiang Yixue Zazhi West China J. Stomatol.* **35**, 155–161 (2017).

218. Sharifi, R. *et al.* Evaluation of Serum and Salivary Iron and Ferritin Levels in Children with Dental Caries: A Meta-Analysis and Trial Sequential Analysis. *Children* **8**, 1034 (2021).

219. Granja, G. L. *et al.* Occurrence of bruxism in individuals with autism spectrum disorder: A systematic review and meta‐analysis. *Spec. Care Dentist.* **42**, 476–485 (2022).

220. Xu, S. *et al.* The association between periodontal disease and the risk of myocardial infarction: a pooled analysis of observational studies. *BMC Cardiovasc. Disord.* **17**, 50 (2017).

221. Zeng, L.-N. *et al.* Oral health in patients with stroke: a meta-analysis of comparative studies. *Top. Stroke Rehabil.* **27**, 75–80 (2020).

222. Hatipoğlu, Ö. & Pertek Hatipoğlu, F. Association between asthma and caries-related salivary factors: a meta-analysis. *J. Asthma Off. J. Assoc. Care Asthma* **59**, 38–53 (2022).

223. Arduino, P. G., Cabras, M., Lodi, G. & Petti, S. Herpes simplex virus type 1 in subgingival plaque and periodontal diseases. Meta‐analysis of observational studies. *J. Periodontal Res.* **57**, 256–268 (2022).

224. Lianhui, Y., Meifei, L., Zhongyue, H. & Yunzhi, F. [Association between chronic periodontitis and hyperlipidemia: a Meta-analysis based on observational studies]. *Hua Xi Kou Qiang Yi Xue Za Zhi Huaxi Kouqiang Yixue Zazhi West China J. Stomatol.* **35**, 419–426 (2017).

225. Shi, T. *et al.* Periodontal disease and susceptibility to breast cancer: A meta-analysis of observational studies. *J. Clin. Periodontol.* **45**, 1025–1033 (2018).

226. Zeng, X.-T. *et al.* Periodontal Disease and Risk of Head and Neck Cancer: A Meta-Analysis of Observational Studies. *PLoS ONE* **8**, e79017 (2013).

227. Zeng, L. *et al.* Oral health in patients with dementia: A meta‐analysis of comparative and observational studies. *Int. J. Geriatr. Psychiatry* **36**, 467–478 (2021).

228. Zeng, X.-T. *et al.* Periodontal Disease and Incident Lung Cancer Risk: A Meta-Analysis of Cohort Studies. *J. Periodontol.* **87**, 1158–1164 (2016).

229. Sgolastra, F., Petrucci, A., Severino, M., Gatto, R. & Monaco, A. Relationship between Periodontitis and Pre-Eclampsia: A Meta-Analysis. *PLoS ONE* **8**, e71387 (2013).

230. Uppal, A. *et al.* The Effectiveness of Periodontal Disease Treatment During Pregnancy in Reducing the Risk of Experiencing Preterm Birth and Low Birth Weight. *J. Am. Dent. Assoc.* **141**, 1423–1434 (2010).

231. Huang, X. *et al.* Maternal periodontal disease and risk of preeclampsia: A meta-analysis. *J. Huazhong Univ. Sci. Technolog. Med. Sci.* **34**, 729–735 (2014).

232. Liew, A., Punnanithinont, N., Lee, Y.-C. & Yang, J. Effect of non-surgical periodontal treatment on HbA1c: a meta-analysis of randomized controlled trials. *Aust. Dent. J.* **58**, 350–357 (2013).

233. Xuan, K., Jha, A. R., Zhao, T., Uy, J. P. & Sun, C. Is periodontal disease associated with increased risk of colorectal cancer? A meta‐analysis. *Int. J. Dent. Hyg.* **19**, 50–61 (2021).

234. Ren, H. G. *et al.* Oral health and risk of colorectal cancer: results from three cohort studies and a meta-analysis. *Ann. Oncol.* **27**, 1329–1336 (2016).

235. Xu, S., Zhang, G., Xia, C. & Tan, Y. Associations Between Poor Oral Health and Risk of Squamous Cell Carcinoma of the Head and Neck: A Meta-Analysis of Observational Studies. *J. Oral Maxillofac. Surg.* **77**, 2128–2142 (2019).

236. Qiu-Ying Sun *et al.* Effects of Periodontal Treatment on Glycemic Control in Type 2 Diabetic Patients: A Meta-Analysis of Randomized Controlled Trials. *Chin. J. Physiol.* **57**, (2014).

237. George, A. *et al.* Periodontal treatment during pregnancy and birth outcomes: a meta-analysis of randomised trials. *Int. J. Evid. Based Healthc.* **9**, 122–147 (2011).

238. Michaud, D. S., Fu, Z., Shi, J. & Chung, M. Periodontal Disease, Tooth Loss, and Cancer Risk. *Epidemiol. Rev.* **39**, 49–58 (2017).

239. Zhong, H.-J., Xie, H.-X., Luo, X.-M. & Zhang, E.-H. Association between periodontitis and systemic lupus erythematosus: a meta-analysis. *Lupus* **29**, 1189–1197 (2020).

240. Le, Q.-A. *et al.* DIFFERENTIAL IMPACT OF PERIODONTAL TREATMENT STRATEGIES DURING PREGNANCY ON PERINATAL OUTCOMES: A SYSTEMATIC REVIEW AND META-ANALYSIS. *J. Evid.-Based Dent. Pract.* **22**, 101666 (2022).

241. Noites, R., Teixeira, M., Cavero-Redondo, I., Alvarez-Bueno, C. & Ribeiro, F. Apical Periodontitis and Cardiovascular Disease in Adults: A Systematic Review with Meta-Analysis. *Rev. Cardiovasc. Med.* **23**, 0100 (2022).

242. Ahmadinia, A. R. *et al.* Association between type 2 diabetes (T2D) and tooth loss: a systematic review and meta-analysis. *BMC Endocr. Disord.* **22**, 100 (2022).

243. Sgolastra, F., Severino, M., Pietropaoli, D., Gatto, R. & Monaco, A. Effectiveness of Periodontal Treatment to Improve Metabolic Control in Patients With Chronic Periodontitis and Type 2 Diabetes: A Meta-Analysis of Randomized Clinical Trials. *J. Periodontol.* **84**, 958–973 (2013).

244. Ye, L., Jiang, Y., Liu, W. & Tao, H. Correlation between periodontal disease and oral cancer risk: A meta-analysis. *J. Cancer Res. Ther.* **12**, 237 (2016).

245. Polyzos, N. P. *et al.* Effect of periodontal disease treatment during pregnancy on preterm birth incidence: a metaanalysis of randomized trials. *Am. J. Obstet. Gynecol.* **200**, 225–232 (2009).

246. Wei, B.-J., Chen, Y.-J., Yu, L. & Wu, B. Periodontal Disease and Risk of Preeclampsia: A Meta-Analysis of Observational Studies. *PLoS ONE* **8**, e70901 (2013).

247. Yao, Q.-W., Zhou, D.-S., Peng, H.-J., Ji, P. & Liu, D.-S. Association of periodontal disease with oral cancer: a meta-analysis. *Tumor Biol.* **35**, 7073–7077 (2014).

248. Zhu, C. *et al.* Association between Herpesviruses and Chronic Periodontitis: A Meta-Analysis Based on Case-Control Studies. *PLOS ONE* **10**, e0144319 (2015).

249. Ma, P., Dai, S., Jin, C., Yao, Y. & Zou, C. Tooth loss and risk of colorectal cancer: a dose&ndash;response meta-analysis of prospective cohort studies. *OncoTargets Ther.* **Volume 11**, 1617–1623 (2018).

250. Li, F. *et al.* Herpesviruses in etiopathogenesis of aggressive periodontitis: A meta-analysis based on case-control studies. *PLOS ONE* **12**, e0186373 (2017).

251. Qin, X., Zhao, Y. & Guo, Y. Periodontal disease and myocardial infarction risk: A meta-analysis of cohort studies. *Am. J. Emerg. Med.* **48**, 103–109 (2021).

252. Zeng, X.-T. *et al.* Periodontal Disease and Risk of Chronic Obstructive Pulmonary Disease: A Meta-Analysis of Observational Studies. *PLoS ONE* **7**, e46508 (2012).

253. Zhang, Y. *et al.* Is periodontitis a risk indicator for gastrointestinal cancers? A meta‐analysis of cohort studies. *J. Clin. Periodontol.* **47**, 134–147 (2020).

254. Wang, T.-F., Jen, I.-A., Chou, C. & Lei, Y.-P. Effects of Periodontal Therapy on Metabolic Control in Patients With Type 2 Diabetes Mellitus and Periodontal Disease: A Meta-Analysis. *Medicine (Baltimore)* **93**, e292 (2014).

255. Liu, F. *et al.* A meta-analysis of emotional disorders as possible risk factors for chronic periodontitis. *Medicine (Baltimore)* **97**, e11434 (2018).

256. Rodrigues, R. P. C. B. *et al.* Salivary changes in chronic kidney disease and in patients undergoing hemodialysis: a systematic review and meta-analysis. *J. Nephrol.* **35**, 1339–1367 (2022).

257. Chen, Z. *et al.* A meta-analysis of the association between the presence of Helicobacter pylori and periodontal diseases. *Medicine (Baltimore)* **98**, e15922 (2019).

258. Liu, Z. *et al.* Systemic Oxidative Stress Biomarkers in Chronic Periodontitis: A Meta-Analysis. *Dis. Markers* **2014**, 1–10 (2014).

259. She, Y. *et al.* Periodontitis and inflammatory bowel disease: a meta-analysis. *BMC Oral Health* **20**, 67 (2020).

260. Zeng, X.-T. *et al.* Periodontal disease and carotid atherosclerosis: A meta-analysis of 17,330 participants. *Int. J. Cardiol.* **203**, 1044–1051 (2016).

261. Gao, Z., Lv, J. & Wang, M. Epstein–Barr virus is associated with periodontal diseases: A meta-analysis based on 21 case–control studies. *Medicine (Baltimore)* **96**, e5980 (2017).

262. Khodadadi, N., Khodadadi, M. & Zamani, M. Is periodontitis associated with obstructive sleep apnea? A systematic review and meta-analysis. *J. Clin. Exp. Dent.* e359–e365 (2022) doi:10.4317/jced.59478.

263. Foratori-Junior, G. A. *et al.* Is overweight associated with periodontitis in pregnant women? Systematic review and meta-analysis. *Jpn. Dent. Sci. Rev.* **58**, 41–51 (2022).

264. Zhang, S. *et al.* Oral manifestations of patients with systemic sclerosis: a meta-analysis for case-controlled studies. *BMC Oral Health* **21**, 250 (2021).

265. Ma, H., Zheng, J. & Li, X. Potential risk of certain cancers among patients with Periodontitis: a supplementary meta-analysis of a large-scale population. *Int. J. Med. Sci.* **17**, 2531–2543 (2020).

266. Sun, J. *et al.* Non-surgical periodontal treatment improves rheumatoid arthritis disease activity: a meta-analysis. *Clin. Oral Investig.* **25**, 4975–4985 (2021).

267. Li, Q. *et al.* Effect of non-surgical periodontal treatment on glycemic control of patients with diabetes: a meta-analysis of randomized controlled trials. *Trials* **16**, 291 (2015).

268. López-Valverde, N. *et al.* Possible Association of Periodontal Diseases With Helicobacter pylori Gastric Infection: A Systematic Review and Meta-Analysis. *Front. Med.* **9**, 822194 (2022).

269. Wei, X. *et al.* The association between chronic periodontitis and oral Helicobacter pylori: A meta-analysis. *PLOS ONE* **14**, e0225247 (2019).

270. Gao, S. *et al.* Periodontitis and Number of Teeth in the Risk of Coronary Heart Disease: An Updated Meta-Analysis. *Med. Sci. Monit.* **27**, (2021).

271. Zheng, M. *et al.* Prevalence of periodontitis in people clinically diagnosed with diabetes mellitus: a meta-analysis of epidemiologic studies. *Acta Diabetol.* **58**, 1307–1327 (2021).

272. Guo, H. *et al.* The Effect of Periodontitis on Dementia and Cognitive Impairment: A Meta-Analysis. *Int. J. Environ. Res. Public. Health* **18**, 6823 (2021).

273. Qiao, P. *et al.* Psoriasis Patients Suffer From Worse Periodontal Status—A Meta-Analysis. *Front. Med.* **6**, 212 (2019).

274. Huang, Y. *et al.* Effects of non-surgical periodontal therapy on periodontal clinical data in periodontitis patients with rheumatoid arthritis: a meta-analysis. *BMC Oral Health* **21**, 340 (2021).

275. Xu, S., Zhang, G., Guo, J. & Tan, Y. Associations between osteoporosis and risk of periodontitis: A pooled analysis of observational studies. *Oral Dis.* **27**, 357–369 (2021).

276. Maulani, C. *et al.* Association between Epstein-Barr virus and periodontitis: A meta-analysis. *PLOS ONE* **16**, e0258109 (2021).

277. Leng, W.-D., Zeng, X.-T., Kwong, J. S. W. & Hua, X.-P. Periodontal disease and risk of coronary heart disease: An updated meta-analysis of prospective cohort studies. *Int. J. Cardiol.* **201**, 469–472 (2015).

278. Zhang, J., Jiang, H., Sun, M. & Chen, J. Association between periodontal disease and mortality in people with CKD: a meta-analysis of cohort studies. *BMC Nephrol.* **18**, 269 (2017).

279. Shao, J. *et al.* Periodontal Disease and Breast Cancer: A Meta-Analysis of 1,73,162 Participants. *Front. Oncol.* **8**, 601 (2018).

280. Shi, Q. *et al.* Patients with Chronic Obstructive Pulmonary Disease Suffer from Worse Periodontal Health—Evidence from a Meta-Analysis. *Front. Physiol.* **9**, 33 (2018).

281. Qiu, C., Zhou, W., Shi, W. & Song, Z. Association between periodontitis and Alzheimer disease: a meta analysis. *Shanghai J. Stomatol.* **29**, 661–668 (2020).

282. Xie, W.-Z. *et al.* Periodontal Disease and Risk of Bladder Cancer: A Meta-Analysis of 298476 Participants. *Front. Physiol.* **9**, 979 (2018).

283. Wu, Z., Xiao, C., Chen, F., Wang, Y. & Guo, Z. Pulmonary disease and periodontal health: a meta-analysis. *Sleep Breath.* (2022) doi:10.1007/s11325-022-02577-3.

284. Chen, J. *et al.* Tooth Loss Is Associated With Increased Risk of Dementia and With a Dose-Response Relationship. *Front. Aging Neurosci.* **10**, 415 (2018).

285. Sayeed, G. & Varghese, S. Association between periodontitis and metabolic syndrome in females: A systematic review and meta-analysis. *J. Int. Soc. Prev. Community Dent.* **0**, 0 (2021).

286. França, L. F. de C. *et al.* Comparative analysis of blood parameters of the erythrocyte lineage between patients with chronic periodontitis and healthy patients: Results obtained from a meta-analysis. *Arch. Oral Biol.* **97**, 144–149 (2019).

287. Shi, Q. *et al.* Association between Myocardial Infarction and Periodontitis: A Meta-Analysis of Case-Control Studies. *Front. Physiol.* **7**, (2016).

288. Wu, S.-Y. *et al.* Periodontal conditions in patients with Sjögren’s syndrome: A meta-analysis. *J. Dent. Sci.* **16**, 1222–1232 (2021).

289. Simpson, T. C. *et al.* Treatment of periodontitis for glycaemic control in people with diabetes mellitus. *Cochrane Database Syst. Rev.* **2022**, (2022).

290. Luo, Y. *et al.* Effect of periodontal treatments on blood pressure. *Cochrane Database Syst. Rev.* **2021**, (2021).

291. Irwandi, R. A., Kuswandani, S. O., Harden, S., Marletta, D. & D’Aiuto, F. Circulating inflammatory cell profiling and periodontitis: A systematic review and meta‐analysis. *J. Leukoc. Biol.* **111**, 1069–1096 (2022).

292. Antonarakis, G. S., Palaska, P.-K. & Herzog, G. Caries prevalence in non-syndromic patients with cleft lip and/or palate: a meta-analysis. *Caries Res.* **47**, 406–413 (2013).

293. Grewcock, R. E., Innes, N. P. T., Mossey, P. A. & Robertson, M. D. Caries in children with and without orofacial clefting: A systematic review and meta-analysis. *Oral Dis.* **28**, 1400–1411 (2022).
